# A neural signature of sleep deprivation in the human brain

**DOI:** 10.1038/s41467-026-75661-x

**Published:** 2026-07-22

**Authors:** Zhenfu Wen, Edward F. Pace-Schott, Peter L. Franzen, Lihan Cui, Kai Zhang, Si Gao, L. Elliot Hong, Peter Kochunov, Anne Germain, Mohammed R. Milad

**Affiliations:** 1 Faillace Department of Psychiatry and Behavioral Sciences, McGovern Medical School, University of Texas Health Science Center at Houston, Houston, TX USA; 2 Department of Psychiatry, Mass General Brigham Hospital and Harvard Medical School, Charlestown, MA USA; 3 Athinoula A. Martinos Center for Biomedical Imaging, Massachusetts General Hospital, Charlestown, MA USA; 4 Department of Psychiatry, University of Pittsburgh, Pittsburgh, PA USA; 5 NOCTEM, LLC, Pittsburgh, PA USA; 6 Present Address: Shien-Ming Wu School of Intelligent Engineering, South China University of Technology, Guangzhou, China

**Keywords:** Predictive markers, Human behaviour, Sleep deprivation, Cognitive neuroscience

## Abstract

Insufficient sleep disrupts cognitive and emotional functioning, yet the precise neural consequences of sleep loss and their persistence remain unclear. Here, we leverage machine learning and large neuroimaging datasets to identify a candidate neural signature that robustly distinguishes sleep-deprived from well-rested brains. We validate this signature across multiple independent datasets spanning both controlled experimental and real-world settings. The signature not only detects residual neural disturbances following a night of recovery sleep, but also demonstrates sensitivity to partial sleep deprivation. Additionally, it captures natural variations in sleep duration in the general population, independent of experimental manipulation. We further identify distributed connectivity patterns that contribute to the signature, highlighting networks vulnerable to sleep manipulations and those that are resistant or rapidly normalized after recovery sleep. The reliability and generalizability of this neural signature underscore its potential as a biomarker for understanding and monitoring the neural impacts of acute and chronic sleep loss.

## Abstract (web summary)

Researchers identified a brain connectivity pattern that reliably reflects sleep deprivation. The pattern generalized across multiple independent datasets and was associated with both experimental sleep loss and natural variation in sleep duration.

## Introduction

Sleep is essential for normal cognitive and emotional functioning. Disturbed or insufficient sleep leads to deficits in attention, learning and memory, emotion processing, and many other functions that underlie daily functioning, productivity, and mental health^1–5^. The sleep loss-related functional deficits and their underlying neural mechanisms have been widely studied using laboratory-based sleep deprivation paradigms^6–8^.

Across various cognitive and emotional tasks, sleep deprivation has been associated with alterations in activation and connectivity of the amygdala, prefrontal cortex, and other regions involved in cognitive control and emotional regulation^8–10^. Compared to well-rested brains, sleep-deprived brains also exhibit abnormal connectivity in intrinsic networks such as the default mode network and attention-related networks^11–13^. Despite extensive research examining the neural impacts of sleep deprivation, most studies have used conventional univariate approaches to test group differences (i.e., sleep-deprived versus well-rested groups). Such studies have focused on isolated regional activations or specific inter-regional connections, leaving less understood the integrative neural effects of sleep deprivation^14–16^, and how these effects are restored after recovery sleep. However, it is increasingly recognized that distributed neural circuits are integrated to enable mental processes^17–19^. Given the profound impacts of sleep deprivation on diverse functions, large-scale neural systems and their interactions are likely disrupted in sleep-deprived brains. Therefore, a comprehensive investigation of functional connectivity at the whole-system level is essential for a thorough understanding of the neural consequences of acute or chronic sleep loss.

Sleep deprivation studies have long suffered from limited statistical power due to small sample sizes^20^, leading to high risk of type I and type II errors and a lack of replication across studies^21–24^. For example, disruption between the default mode and attention-related networks, which was considered one of the most robust effects of sleep deprivation, was inconsistently reported across studies^25^. Additionally, given inter-individual variability, traditional group-level inference methods provide insufficient information about individual differences in neural responses to sleep loss. Recent advances in individualized neuroscience emphasize the importance of moving beyond group-level analyses to characterize brain-behavior relationships at an individual level^26–28^. Estimations of the neural impacts of sleep deprivation at an individual level would potentially enhance our understanding of the heterogeneous responses to sleep disturbance and inform objective biomarker developments aimed at sleep health monitoring and interventions.

A powerful methodology for inference at the individual level is machine learning-based multivariate prediction^29–32^, which integrates neural patterns across distributed circuits such as whole-brain connectivity measures to construct predictive models that distinguish between different mental states. The resulting models, often referred to as decoders or neural signatures, offer higher sensitivity and reproducibility compared to traditional univariate analyses^21,33,34^. Using multivariate prediction techniques, recent studies have predicted individual differences in sleep quantity and/or quality from large-scale neuroimaging datasets^35,36^. These works have significantly advanced the understanding of how large-scale neural organization reflects individual variation in sleep behavior. However, most of these studies have been linking brain measures to spontaneous variability in sleep rather than experimentally manipulating sleep. This makes it difficult to isolate the direct neural consequences of acute sleep loss from other factors that may co-vary with habitual sleep duration or quality.

Building on this progress, we aimed to extend multivariate prediction approaches to controlled sleep manipulation paradigms, which allow direct characterization of the neural consequences of acute sleep loss while minimizing individual confounds through random group assignment to sleep conditions. A key advantage of predictive modeling is that the constructed decoders can generalize across contexts^24,32^, enabling the evaluation of neural signature expression under varying conditions, such as sleep deprivation, recovery sleep, or habitual sleep. In the case of sleep deprivation, such a signature may capture both transient disruptions induced by sleep loss in neural systems and the residual perturbations that persist after recovery sleep, providing insights into the dynamic effects of sleep manipulation. However, to date, a sensitive and generalizable neural signature of sleep deprivation has yet to be established.

Here, we extend recent studies linking brain connectivity and sleep to experimentally manipulated sleep paradigms, isolating the neural consequences of acute sleep loss. Leveraging large neuroimaging datasets (Table 1), we develop and validate a neural signature that captures distributed neural impacts of sleep deprivation. Our objectives are to (i) evaluate the generalizability of the neural signature in distinguishing sleep-deprived brains from well-rested ones; (ii) examine the responsiveness of the neural signature to other sleep manipulations, such as partial sleep deprivation, and its sensitivity to natural variations in sleep duration; (iii) characterize the distributed connectivity patterns underlying the neural signature, and assess how these patterns are modulated by varying doses of sleep manipulation and restored following recovery sleep.

**Table 1:** Key characteristics of datasets used in the study

Dataset	Role in study	Design type	Sample characteristics	Scanning protocol	Sleep manipulation	Analysis objective
Dataset 1	Model development	Randomized experimental (between-subject)	Sample: 151 healthy adults (69 male, age: 23.8 ± 3.3);	About 26 minutes task-based fMRI.	Randomized: normal sleep (*n* = 48), sleep deprivation (*n* = 52), sleep restriction (*n* = 51).	Train classifier to distinguish sleep deprivation vs. normal sleep
Dataset 2	External validation	Within-subject cross-over	Sample: 68 adults, including 30 major depression disorder (seven male, age: 34 ± 9.9) and 38 healthy control (21 male, 33.5 ± 8.6)	About 7 min resting-state fMRI from each session.	Two sessions: normal sleep vs. sleep deprivation	Classify sleep condition within individuals
Dataset 3	External validation	Within-subject cross-over	Sample: 54 adults (23 male, age: 30.6 ± 9.4), including 44 patients with treatment resistant depression and 10 healthy control.	About 23 min, combined task-based and resting-state fMRI from each session.	Two sessions: normal sleep vs. sleep deprivation	Classify sleep condition within individuals
Dataset 4	External validation	Within-subject cross-over	Sample: 78 healthy adults, including 42 younger (21 male, age: 20–30 years) and 36 older volunteers (17 male, age: 65–75 years).	About 16 minutes resting-state fMRI from each session.	Two sessions: normal sleep vs. sleep restriction	Classify sleep condition within individuals
Dataset 5	External validation	Cross-sectional	Sample: 109 adults (40 male; age: 24.7 ± 5.0 years)	About 26 minutes task-based fMRI from one session.	No manipulation; Total sleep time from actigraphy ( ~ 2 weeks).	Correlate probability of being sleep-deprived (PoSD) with habitual sleep duration.
Dataset 6	External validation	Cross-sectional	Sample: 39,799 adults (age: 40–69)	About 6 min. resting-state fMRI from one session.	No manipulation; Total sleep time estimated from self-report and accelerometry.	Correlate PoSD with habitual sleep duration.

## Results

### Neural signature of sleep deprivation

**Fig. 1:**
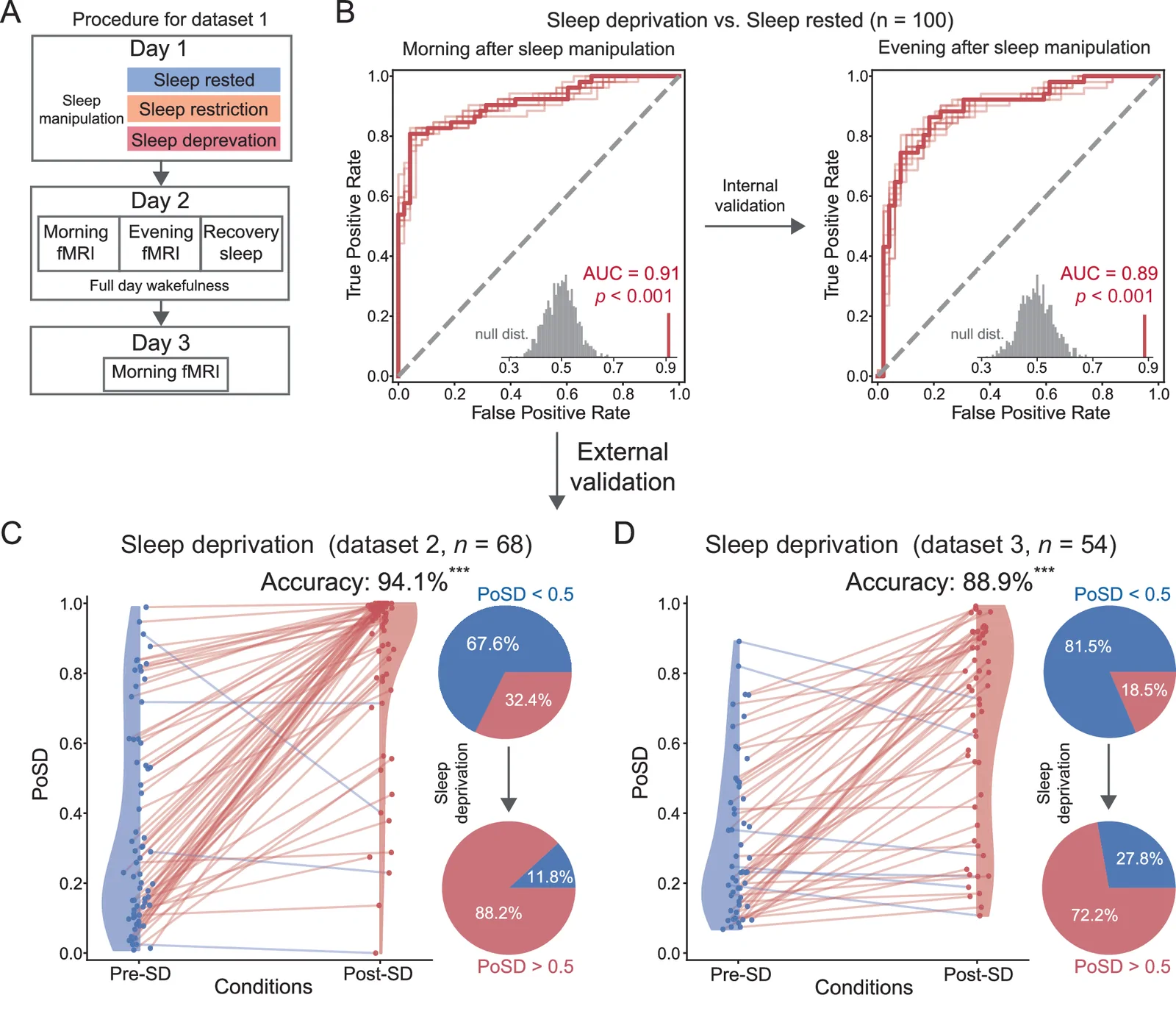
Predictive performance of the neural signature of sleep deprivation. **A** Experimental procedure for the discovery dataset. **B** Classification performance on the discovery dataset in discriminating sleep-deprived brains from rested brains (*n* = 100). Left: Cross-validation results on data from the morning session after sleep deprivation (SD). Right: Applying model trained on data from the morning session to test data from the evening session on the same day. Each receiver operating characteristic (ROC) curve represents a repetition of the cross-validation procedure. The bolded ROC curve represents the mean across 10 repetitions. Area under the ROC curve (AUC) significance was assessed against chance level (AUC = 0.5) using two-sided permutation tests with 1,000 permutations; none of the permuted AUC values exceeded the observed AUC in either case (*p* < 0.001). **C**,**D** Generalization on dataset 2 (**C**, *n* = 68) and dataset 3 (**D**, *n* = 54) in discriminating sleep-deprived brains from rested brains. Each dot indicates an individual. A line connects dots of the same individual, and the color of a line indicates whether an individual’s probability of being sleep-deprived (PoSD) score is increased (red) or decreased (blue) after SD. Differences in PoSD between rested and sleep-deprived sessions were assessed using two-sided paired t-tests (dataset 2: *p* = 1.36E-19; dataset 3: *p* = 1.80E-12). Forced-choice classification accuracy was evaluated using two-sided permutation tests with 1000 permutations (*p* < 0.001 for both datasets). ***: *p* < 0.001. Source data are provided as a Source Data file.

We constructed a decoder to discriminate sleep-deprived individuals from well-rested individuals based on whole-brain functional connectivity. The model construction was based on a discovery dataset (dataset 1, *n* = 151), which included healthy adult participants who maintained regular sleep/wake schedules and obtained sufficient sleep prior to attending the study (Methods). The participants were randomly assigned to be awake for a whole night (sleep deprivation group, *n* = 52), receive a night of normal sleep (normal sleep group, *n* = 48), or receive only half night of sleep (sleep restriction group, *n* = 51). All participants underwent three task-based fMRI sessions, a morning session (26 minutes of fMRI data) and an evening session (32 min of fMRI data) following sleep manipulation, and a morning session (32 min of fMRI data) the next day after recovery sleep (Fig. 1A, Methods). All participants completed identical task structures with matched timing and composition across conditions, minimizing potential confounds from task differences. In addition, part of the paradigm involved passive stimulus exposure, reducing performance-related variability. The time series of 442 brain regions across the whole brain, including 400 cortical regions^37^, 32 subcortical regions^38^, and 10 cerebellum regions^39^, were extracted and used for estimating interregional functional connections for each participant. These 97,461 connections were used as features (without feature selection) for a linear kernel support vector machine (SVM; default hyperparameter: *C* = 1.0). Given the expected larger neural effects of sleep deprivation compared to sleep restriction, we focused on discriminating the sleep deprivation group from the normal sleep group, and left the sleep restriction group for validation (see below).

We evaluated the classification performance in the morning following sleep manipulation using a 10 × 10 cross-validation procedure. Specifically, participants were randomly partitioned into ten folds; in each iteration, one fold was held out as the test set while the remaining nine folds were used for model training. This cross-validation procedure was repeated ten times with different random splits, and the mean performance across all ten repetitions was reported. The constructed neural signature accurately distinguished sleep-deprived individuals from well-rested individuals (area under the curve [AUC] = 0.91, Fig. 1B), which is significantly higher than chance level (AUC = 0.5) based on a permutation test (*p* < 0.001). The performance was insensitive to methodological variation as similar performance was obtained without the use of global signal regression in the preprocessing workflow (Supplementary Fig. 1). We further compared the whole-brain model with models based on connectivity within individual canonical networks. These network-restricted models showed variable performance (AUC = 0.62–0.85, Supplementary Fig. 2) that was generally lower than the whole-brain model (AUC = 0.91), suggesting that integrating distributed connectivity provides improved predictive performance. We then validated the whole-brain model performance within the same cohort by applying the model to data collected in the evening after sleep manipulation. This analysis resulted in similar performance (AUC = 0.89, *p* < 0.001, Fig. 1B), indicating internal robustness of the neural signature.

### Generalization of the neural signature

Next, we tested the generalizability of this neural signature by applying it to independent participants whose data had never been used in model training. A final decoder was retrained on all participants from the discovery dataset (sleep-deprivation and normal-sleep groups) and applied directly to the independent validation datasets without further modification. The constructed model provided a single score—the probability of being sleep-deprived (PoSD), for each participant, reflecting the model’s relative confidence that an individual exhibits connectivity patterns associated with sleep deprivation. The PoSD was estimated using a Platt scaling procedure to convert the decision values of the SVM classifier into probabilities (range from 0 to 1). Compared to binary class assignments (e.g., sleep-deprived or rested), PoSD provides a probabilistic estimate of sleep deprivation, enabling more granular analyses of individual differences and capturing subtle neural disruptions. A higher PoSD value indicating a higher likelihood of being sleep-deprived.

We validated the model using two independent, publicly available datasets (Table 1). Both datasets employed within-individual cross-over design, enabling the neural signature to be evaluated based on sleep-related state changes in PoSD within the same individual. In addition, scanning times were matched between the normal sleep and sleep deprivation sessions in these datasets, minimizing potential confounding effects of time of day. The first validation dataset (dataset 2, *n* = 68) included individuals that were scanned twice during resting-state—pre and post a night of sleep deprivation^40^. Despite differences in scanning protocols, preprocessing strategies, and task contexts between this dataset and the discovery dataset, the decoder successfully distinguished between rested and sleep-deprived states (Fig. 1C). The sleep-deprived state led to significantly higher PoSD values than the rested state (*t*(67) = 12.76, *p* = 1.36E-19, Cohen’s *d* = 1.90). Before sleep deprivation, only 32.4% of individuals had PoSD values above 0.5, which was increased to 88.2% after a night of sleep deprivation. We then used forced-choice test- an approach that is less vulnerable to site effects^29,31,41^, to assess the classification performance. The sleep-deprived state could be accurately discriminated from the rested state with a forced-choice accuracy of 94.1% (*p* < 0.001, permutation test).

We obtained similar results from the Perturbation of the Depression Connectome (PDC) study dataset (dataset 3, *n* = 54, Fig. 1D)^42^, which included participants whose fMRI scans were collected before and after a night of sleep deprivation. The PoSD was significantly higher post- compared to pre-sleep deprivation (*t*(53) = 9.14, *p* = 1.80E-12, Cohen’s *d* = 1.35). Before sleep deprivation, only 18.5% of the participants had PoSD values above 0.5, which was increased to 72.2% following sleep deprivation. The decoder achieved a classification accuracy of 88.9% (*p* < 0.001, permutation test). Compared to univariate analyses, these prediction-based results were more sensitive and robust in detecting the effects of sleep deprivation (Supplementary results and Supplementary Fig. 3). Since datasets 2 and 3 contain both controls and patients with major depression or treatment-resistant depression, we further compared PoSD changes from rested state to sleep-deprived state between controls and patients. We found no significant group differences in dataset 2 (*t*(66) = -1.38, *p* = 0.172; mean±s.d.: controls, 0.42 ± 0.28; patients, 0.53 ± 0.32) and dataset 3 (*t*(52) = 1.69, *p* = 0.098; controls, 0.45 ± 0.16; patients, 0.30 ± 0.27), suggesting that the neural signature of sleep deprivation generalizes across clinical and non-clinical populations. Together, these external validation results demonstrated the high generalizability of the neural signature in distinguishing sleep-deprived from rested brains.

### Limited impact of head motion

A commonly observed phenomenon in sleep deprivation studies is that head motion during scanning is higher in the sleep-deprived state than in the normal sleep state. In the discovery dataset, we also observed significant differences between the sleep deprivation group and normal sleep group in mean framewise displacement (FD) (*t*(98) = 4.05, *p* = 1.04E-4) and the number of usable volumes after censoring (*t*(98) = 3.51, *p* = 6.88e-4). To ensure that classification performance was not confounded by head motion, we performed additional analyses using a motion-matched subset of participants. Specifically, we excluded 10 participants with the highest FD measures from the sleep deprivation group and 10 participants with the lowest FD measures from the normal group, resulting in a subset with comparable motion metrics between groups (total *n* = 80; sleep deprivation: *n* = 42; normal sleep: *n* = 38; FD: *t*(78) = 1.03, *p* = 0.30; usable volumes: *t*(78) = -1.06, *p* = 0.29). We then conducted a 10 × 10 cross-validation procedure on this subset of participants. The resulting classification performance was comparable to the main analysis (AUC = 0.90, permutation test *p* < 0.001).

We further examined potential confounds related to head motion in datasets 3 and 4, for which motion estimates were available. In dataset 3, mean framewise displacement (FD) was comparable between the normal sleep and sleep deprivation sessions (*t*(53) = -1.66, *p* = 0.102), while the number of usable volumes was slightly higher in the sleep deprivation session (*t*(53) = 2.62, *p* = 0.011). To ensure that the observed PoSD differences were not driven by head motion, we used a linear mixed-effects model with sleep condition as a fixed effect and participant as a random effect, while including mean FD and usable volumes as covariates. The effect of sleep condition on PoSD remained significant (*b* = 0.331, *z* = 9.051, *p* = 1.41E-19, 95%*CI* = [0.259, 0.403]). Similar results were observed in dataset 4, where mean FD (*t*(77) = -1.69, *p* = 0.094) and the number of usable volumes (*t*(77) = -0.031, *p* = 0.975) were comparable between normal sleep and sleep deprivation sessions. After controlling for mean FD and usable volumes, the effect of sleep condition on PoSD remained significant (*b* = 0.110, *z* = 3.500, *p* = 4.66E-4, 95%*CI* = [0.048, 0.172]). These results indicate that model predictions primarily reflect sleep condition rather than head motion.

### Expression of the signature under other sleep manipulations

**Fig. 2:**
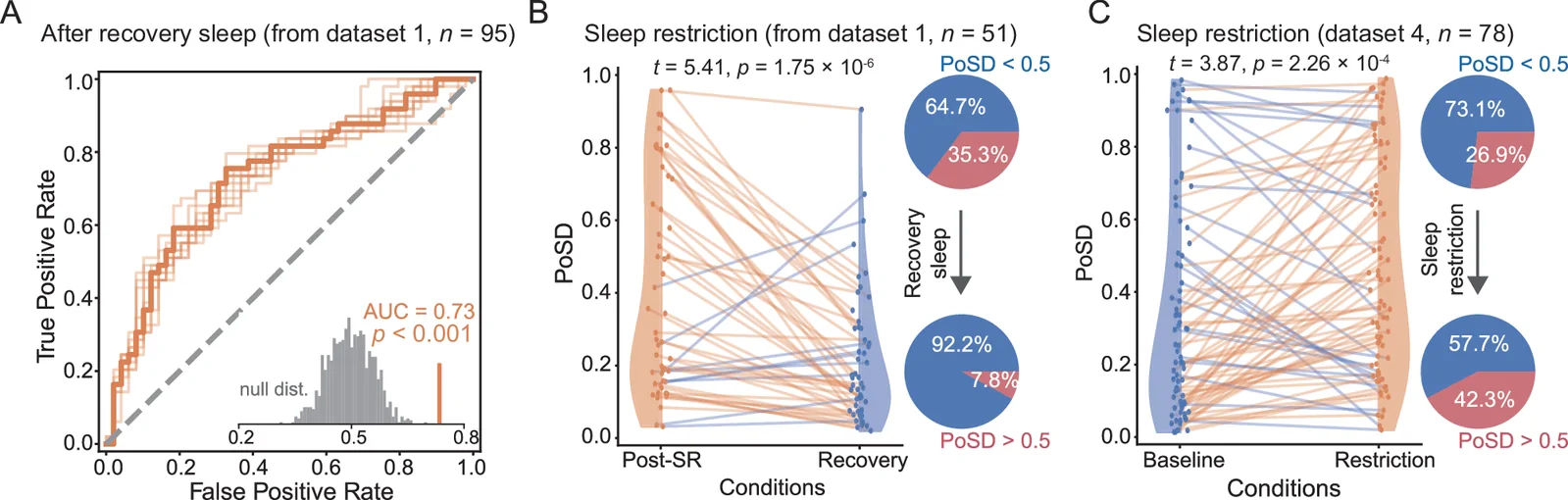
Neural expression of the signature on other sleep conditions. **A** Predictive performance on data from the morning session after a night of recovery sleep (*n* = 95). Each receiver operating characteristic (ROC) curve represents a repetition of the cross-validation procedure. The bolded ROC curve represents the mean across ten repetitions. Area under the ROC curve (AUC) significance was assessed against chance level (AUC = 0.5) using two-sided permutation tests with 1000 permutations; none of the permuted AUC values exceeded the observed AUC (*p* < 0.001). **B** Applying the neural signature to the sleep restriction group from dataset 1 (*n* = 51). For the x-axis, ‘Post-SR’ represents data from the morning session after sleep restriction, ‘Recovery’ represents data from the morning session after a night of recovery sleep. The color of a line indicates whether an individual’s probability of being sleep-deprived (PoSD) score is higher (yellow) or lower (blue) after sleep restriction than after recovery sleep. Differences in PoSD between Post-SR and Recovery were assessed using a two-sided paired t-test (*t*(50) = 5.41, *p* = 1.75E-6). **C** Applying the neural signature to another sleep restriction dataset (dataset 4, *n* = 78). For the x-axis, ‘Baseline’ represents data from the session after normal sleep, ‘Restriction’ represents data from the session after sleep restriction. The color of a line indicates whether an individual’s PoSD score is higher (yellow) or lower (blue) after sleep restriction than at baseline. Differences in PoSD between Baseline and Restriction were assessed using a two-sided paired t-test (*t*(77) = 3.87, *p* = 2.26E-4). Source data are provided as a Source Data file.

We next investigated how the neural signature varied under other sleep conditions that differed from sleep deprivation. First, we examined whether a night of recovery sleep could restore the perturbed brain connectivity. Note that the decoders tested on post-recovery scans were trained on data from independent participants, so that the data of individuals whose recovery scans were tested were excluded from model training. We applied the neural signature to data collected the morning after a recovery night (subsample of dataset 1, normal sleep: *n* = 48, sleep deprivation: *n* = 47), and found that the previously sleep-deprived individuals resulted in significantly higher PoSD than those assigned to the normal sleep group (*t*(93) = 3.94, *p* = 1.64E-4, Cohen’s *d* = 0.81). The decoder significantly distinguished between previously sleep-deprived individuals and rested individuals (AUC = 0.73, *p* < 0.001, permutation test, Fig. 2A). These results suggest that a single night of recovery sleep may not fully restore the functional connectivity perturbations caused by sleep deprivation, indicating the persistence of sleep deprivation effects on brain function.

Next, we evaluated how responsive the neural signature is to milder sleep disturbance, i.e., sleep restriction (remaining awake for half the night). Using data from individuals in the sleep restriction group of dataset 1 (*n* = 51), we tested whether PoSD differed between sleep restriction and recovery sleep. PoSD scores were derived using the final decoder trained only on the sleep-deprivation and normal-sleep groups, ensuring full independence between the training and test data. We found that PoSD scores after sleep restriction were significantly higher than those after a night of recovery sleep (*t*(50) = 5.41, *p* = 1.75E-6, Cohen’s *d* = 0.81, Fig. 2B). Specifically, 35.3% of individuals had PoSD scores higher than 0.5. For descriptive comparison, the corresponding proportions based on cross-validation were 14.6% in the normal sleep group and 82.7% in the sleep deprivation group. This reflects the more moderate impact of sleep restriction than sleep deprivation on brain connectivity. After a night of recovery sleep, this proportion decreased significantly to 7.8%, indicating substantial neural restoration in the sleep-restriction group. We further validated the sensitivity of the signature on individuals from another sleep restriction study (dataset 4, *n* = 78), in which the individuals were well-rested in one session, and were limited to 3 hours of sleep in another session^25,43^. The analysis confirmed a significant increase of PoSD after sleep restriction (post- vs. pre-restriction; *t*(77) = 3.87, *p* = 2.26E-4, Cohen’s *d* = 0.42, Fig. 2C). The moderate effect size on this dataset may relate to its specific experimental design, where participants were provided 3 hours of sleep opportunity right after sleep restriction, which is similar to a recovery sleep. Overall, these findings highlight the sensitivity of the neural signature to varying levels of sleep loss.

### Association with natural sleep variance

**Fig. 3:**
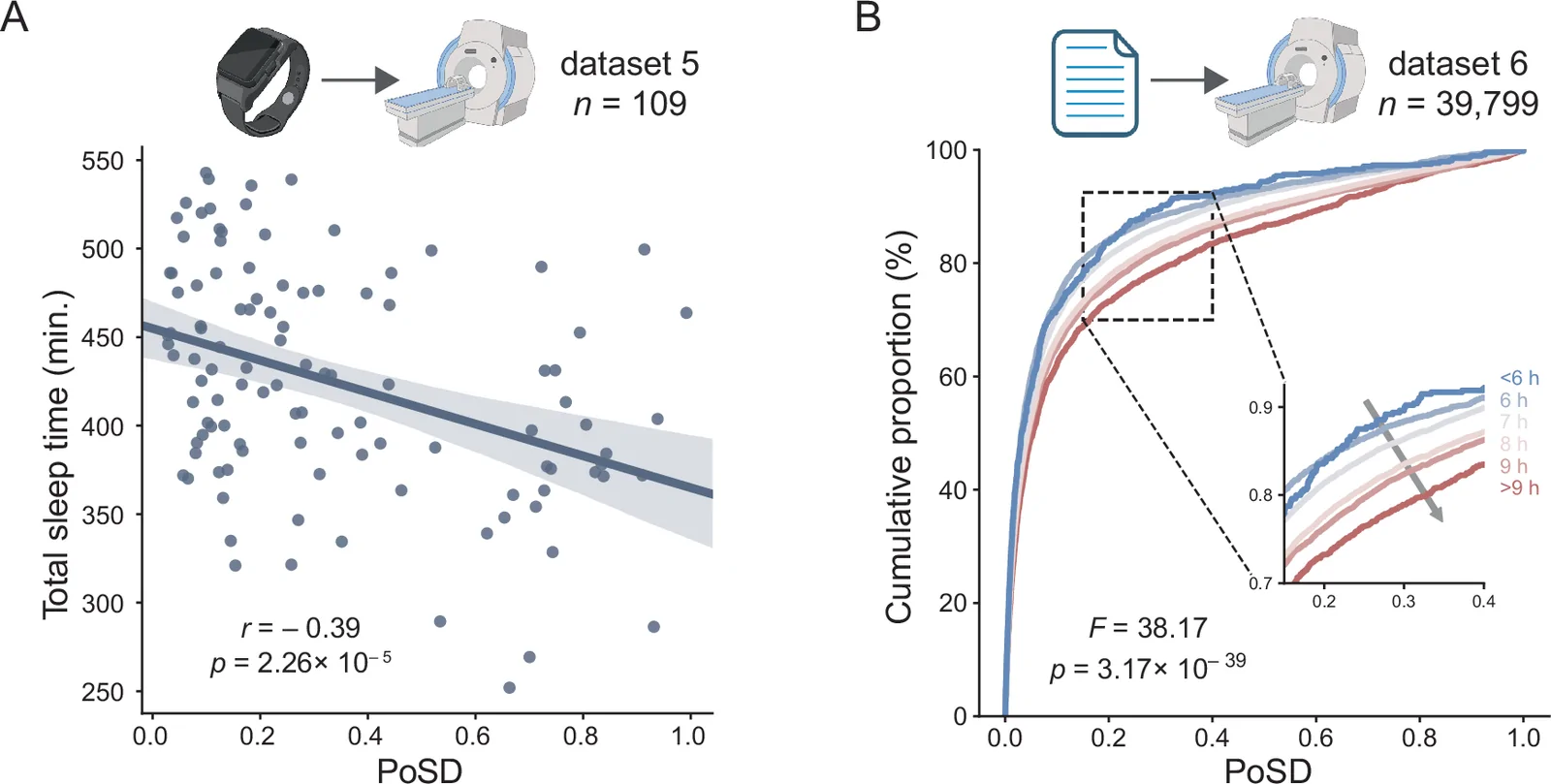
Neural signature associate with habitual sleep duration. **A** The PoSD score negatively correlates with actigraphy-based total sleep time on dataset 5 (*r* = −0.39, *p* = 2.26E-5, two-sided; *n* = 109). The shaded area indicates 95% confidence intervals. **B** The PoSD score is significantly different across groups stratified based on their reported sleep hours on dataset 6 (*F*(5, 39769) = 38.17, *p* = 3.17E-39; *n* = 39,799). PoSD: probability of being sleep-deprived. Icons were created in BioRender, with attribution lines: Zhang, K. (2026) https://BioRender.com/x9nm1pj; Zhang, K. (2026) https://BioRender.com/nogqxly; and Zhang, K. (2026) https://BioRender.com/fuhp8a0. Source data are provided as a Source Data file.

Given that reduced total sleep time (TST) is a key characteristic of sleep deprivation, we hypothesized that the neural signature of sleep deprivation would negatively correlate with TST. To test this, we analyzed data from 109 young adults (dataset 5; age: 24.7 ± 5.0 years; 40 male, 69 female)^44^, who participated in an independent neuroimaging study. Each participant wore a wrist actigraphy device (Actiwatch-2) continuously for two weeks prior to their task-based fMRI session (26 minutes). Actigraphy data were processed using standardized pipelines to derive nightly TST, which was then averaged across all nights with usable data for each participant. As hypothesized, PoSD significantly correlated with TST (*r* = -0.39, *p* = 2.26E-5, 95%*CI*: [-0.54, -0.22], Fig. 3A). The effect remained significant when head motion, age and sex were controlled for (partial correlation, *r* = -0.30, 95%*CI*: [-0.46, -0.12], *p* = 1.74E-3). These results indicated that shorter TSTs are associated with higher expressions of the sleep deprivation neural signature.

We further validated this association on a cohort of UK Biobank participants (dataset 6, *n* = 39,799; age: 63.9 ± 7.7 years; 18,623 male, 21,176 female)^45,46^. On this dataset, PoSD scores were low for most of the participants (median PoSD: 0.04), with 90.7% percent of participants had PoSD lower than 0.5. Note that participants in the UK Biobank were older than those in the discovery dataset (ages 40–69 vs. 18–30), these age-related neural differences may partially contribute to the overall low PoSD scores. Confirming the observation on dataset 5, we found a significant negative correlation between self-reported hours of sleep per night and PoSD (partial correlation, *r* = -0.067, 95%*CI*: [-0.08, -0.06], *p* = 5.28E-41), whilst controlling for covariates including scanning site, movement during scanning, age, and sex. Since the reported sleep hour is a discrete measure, we stratified participants to six groups using their reported TST ( < 6 h, 6 h, 7 h, 8 h, 9 h, and >9 h, much less individuals reported extreme values less than 6 h or more than 9 h, therefore they were combined to two groups here). An analysis of covariance (ANCOVA) with the above-mentioned covariates controlled for, revealed significant differences in PoSD distributions across TST groups (*F*(5, 39769) = 38.17, *p* = 3.17E-39, Fig. 3B). In a subset of participants with their accelerometer-based TST estimation available (n = 17,209; age: 64.0 ± 7.7 years; 7,700 male, 9,509 female)^47^, we further examined the association between PoSD and objectively measured sleep duration. Wrist-worn triaxial accelerometer data were collected continuously for 7 days using the Axivity AX3 device and processed with a machine learning-based pipeline to derive TST, defined as the average proportion of time spent sleeping across the monitoring period. The associations between PoSD and accelerometer-based TST were also significant (partial correlation, *r* = -0.096, 95%*CI*: [-0.11, -0.08], *p* = 3.96E-36, controlled for scanning site, movement during scanning, age, and sex). Considering the out-of-sample prediction nature, overall small effect size in reproducible large-scale brain-wide association studies^21,23^, and the time gap between the accelerometer assessment and neuroimaging session, the effect sizes we observed here were robust and reproducible. These converging results robustly support the hypothesis that the sleep deprivation signature is associated with individual differences in habitual sleep duration.

To evaluate the specificity of PoSD, we examined its associations with additional behavioral and psychological measures in the UK Biobank dataset, including physical activity, cognitive performance, substance use, and adverse childhood experiences (Supplementary Results). Correlations between PoSD and these measures were relatively small ( | *r* | <0.04, Supplementary Table S1), and consistently lower than those observed for total sleep time ( | *r* | = 0.11–0.14). The correlations between PoSD and TST remained significant after controlling for all the other measures ( | *r* | = 0.067-0.096, *p* < 1.60e-36). Together, these results suggest that the neural signature is more strongly associated with sleep-related variation than with other behavioral or cognitive measures.

To further explore the processes reflected by the neural signature, we conducted an exploratory analysis in an independent movie-watching fMRI dataset with continuous ratings of emotional arousal and valence^48,49^. PoSD scores were higher during high-arousal than low-arousal periods, whereas no significant difference was observed between high- and low-valence periods (Supplementary Results). These findings suggest that the neural signature may be more sensitive to arousal-related processes than to affective valence.

### Characterizing the neural signature

**Fig. 4:**
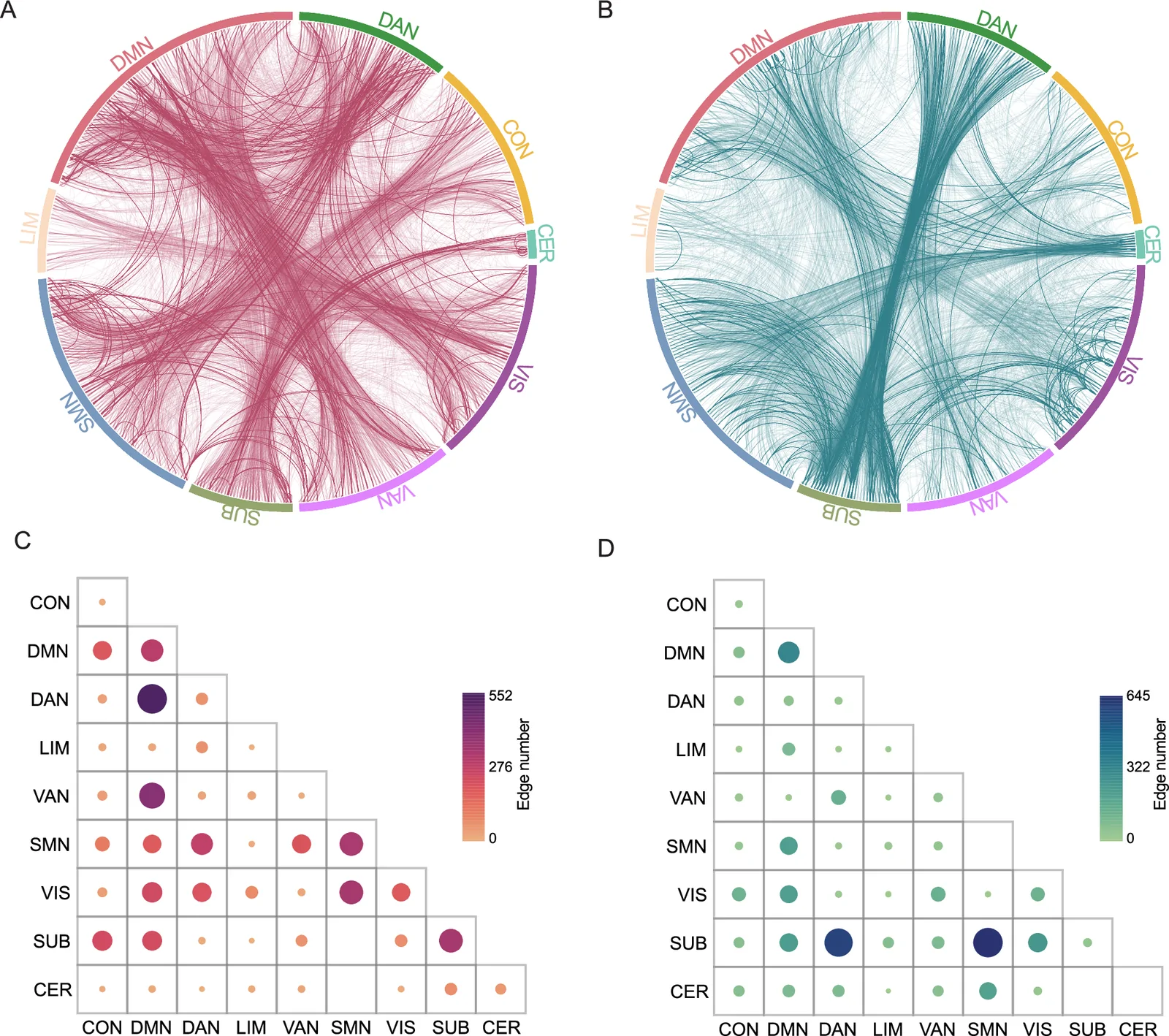
Connections significantly contributing to the neural signature. **A**,**B** Circular plot representation of the connections with positive (**A**) and negative (**B**) predictive weights. Each arc represents a significant connection between the regions at its two ends. The top 5% of connections with the largest predictive patterns are marked with deeper colors. **C**,**D** The number of significant connections with positive (**C**) and negative (**D**) predictive weights within and between the nine networks. Each cell indicates the number of statistically significant connections (based on permutation test, 1000 times) between the corresponding network pairs, indexed on the x- and y-axes. The size and color intensity of each circle are proportional to the number of significant connections. CON frontoparietal control network, DMN default mode network, VAN ventral attention network, DAN dorsal attention network, SMN somatomotor network, VIS visual network, LIM limbic network, SUB subcortical network, CEB cerebellum network. Source data are provided as a Source DataSource Data file.

Having established that the neural signature of sleep deprivation is robust and replicable across independent datasets, we next sought to examine the contributions of different neural systems to this signature. We estimated predictive patterns of the model^50^ and conducted permutation tests (1000 permutations) to identify the connections that significantly contributed to the model^41^. This analysis identified 8600 significant connections (8.8% out of the total 97,461 connections), including 4582 connections (4.7%) with positive predictive patterns (Fig. 4A) and 4018 connections (4.1%) with negative predictive patterns (Fig. 4B). We separated positive versus negative predictive weights to distinguish connections that increased the likelihood of classifying an individual as sleep deprived versus those that increased the likelihood of classifying an individual as well rested. This allowed us to test whether these distinct sets of features were differentially modulated by recovery sleep or partial sleep restriction. These connections were distributed throughout the brain, highlighting the widespread impact of sleep deprivation on functional connectivity.

To better interpret the results, we grouped the brain regions into nine intrinsic neural systems^37–39^: the frontoparietal control network (CON), default mode network (DMN), ventral attention network (VAN), dorsal attention network (DAN), somatomotor network (SMN), visual network (VIS), limbic network (LIM), subcortical network (SUB), and cerebellum network (CEB). We calculated the number of significant connections within and between these networks. For the positive predictive patterns (Fig. 4C), the most prominent contributors are the connections between the DMN and attention networks (DAN and VAN). Conversely, the negative predictive patterns largely involved connections between SUB and other networks, particularly the DAN and SMN (Fig. 4D). Accounting for the different number of regions assigned to the networks, we found that connections involving the CEB also significantly contributed to the signature (Supplementary Fig. 4). For example, connections within CEB significantly contributed to the positive patterns, while connections between CEB and other networks, such as SMN and DAN were significant contributors to the negative patterns.

**Fig. 5:**
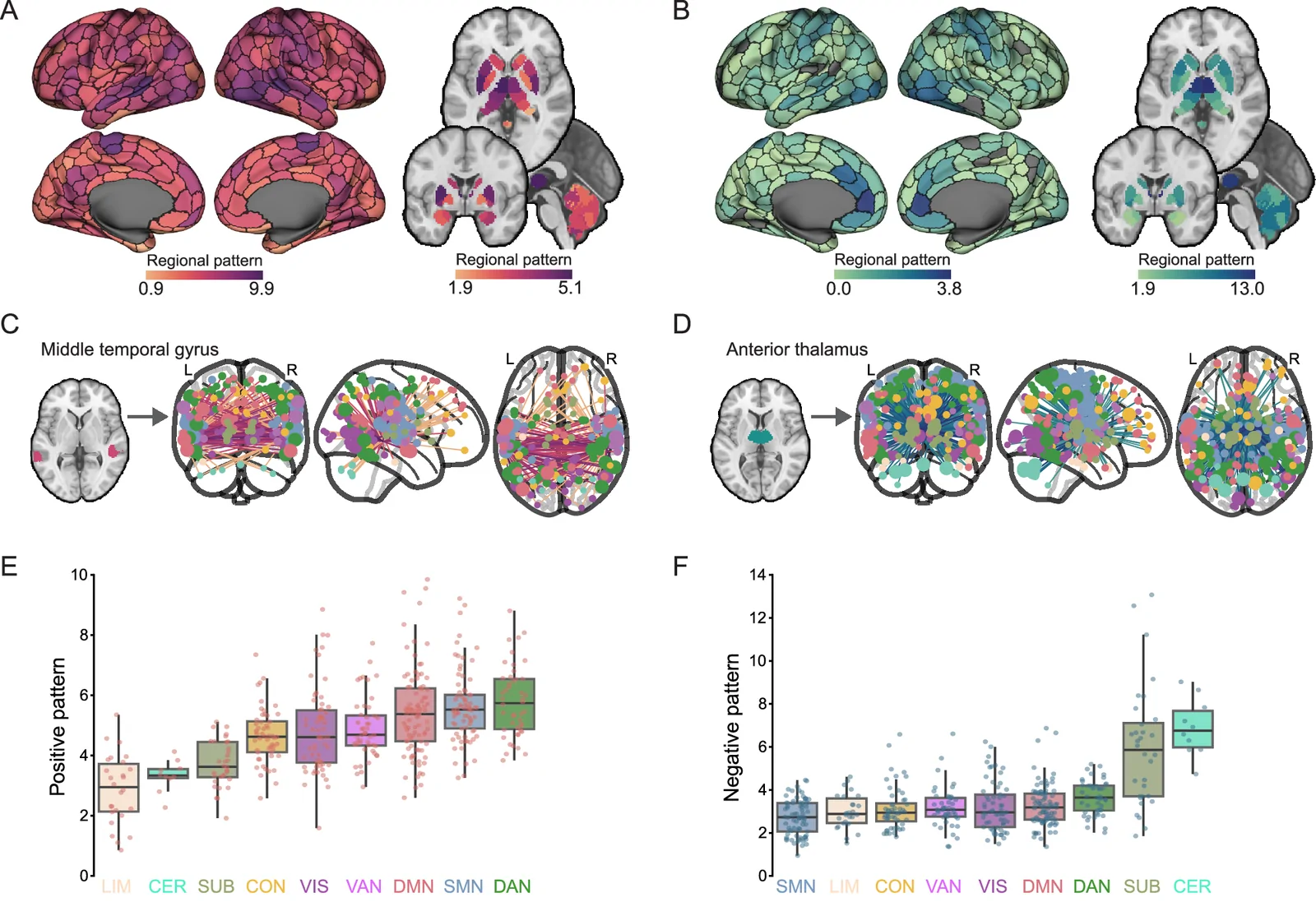
Regional contributions to the neural signature. **A**,**B** Positive (**A**) and negative (**B**) predictive weights for each region. For negative predictive weights, the absolute values were used. **C**,**D** The connections between the regions with the highest positive (**C**) or negative (**D**) regional predictive weight and other regions. Only connections with significant predictive weights were shown. Brain regions were color-coded according to neural system they are assigned to. **E**,**F** Positive (**E**) and negative (**F**) predictive weights for each of the 9 networks. In the box plots, bounds of the box represent the 1st (25%) and 3rd (75%) quartiles, the central line represents the median, the whiskers represent the values within 1.5 times of the interquartile range. Each dot represents a brain region. The number of regions in frontoparietal control (CON), default mode (DMN), dorsal attention (DAN), limbic (LIM), ventral attention (VAN), somatomotor (SMN), visual (VIS), subcortical (SUB), and cerebellum (CER) networks are 52, 91, 46, 26, 47, 77, 61, 32, and 10, respectively. (**A**) and (**B**) were created with Surfplot (v0.2.0) toolbox. (**C**) and (**D**) were created with Nilearn (v0.10.2) toolbox. Source data are provided as a Source Data file.

We further assessed the regional contribution to the signature by separately calculating the sum of significant positive and negative patterns connected with each region. The regions with the highest positive contributions included the middle temporal gyrus (MTG), postcentral gyrus, and regions from the ventral visual cortex (Fig. 5A). The regions with the highest negative contributions included the thalamus, cerebellum, and anterior cingulate cortex (Fig. 5B). Positive and negative predictive patterns connected with the MTG, and the anterior thalamus were visualized, respectively (Fig. 5C and Fig. 5D). Overall, the regions contributing the most to positive patterns were predominantly from DAN, SMN and DMN (Fig. 5E), whereas those contributing the most to negative patterns were largely from CEB and SUB (Fig. 5F).

### Neural patterns under other sleep manipulations

**Fig. 6:**
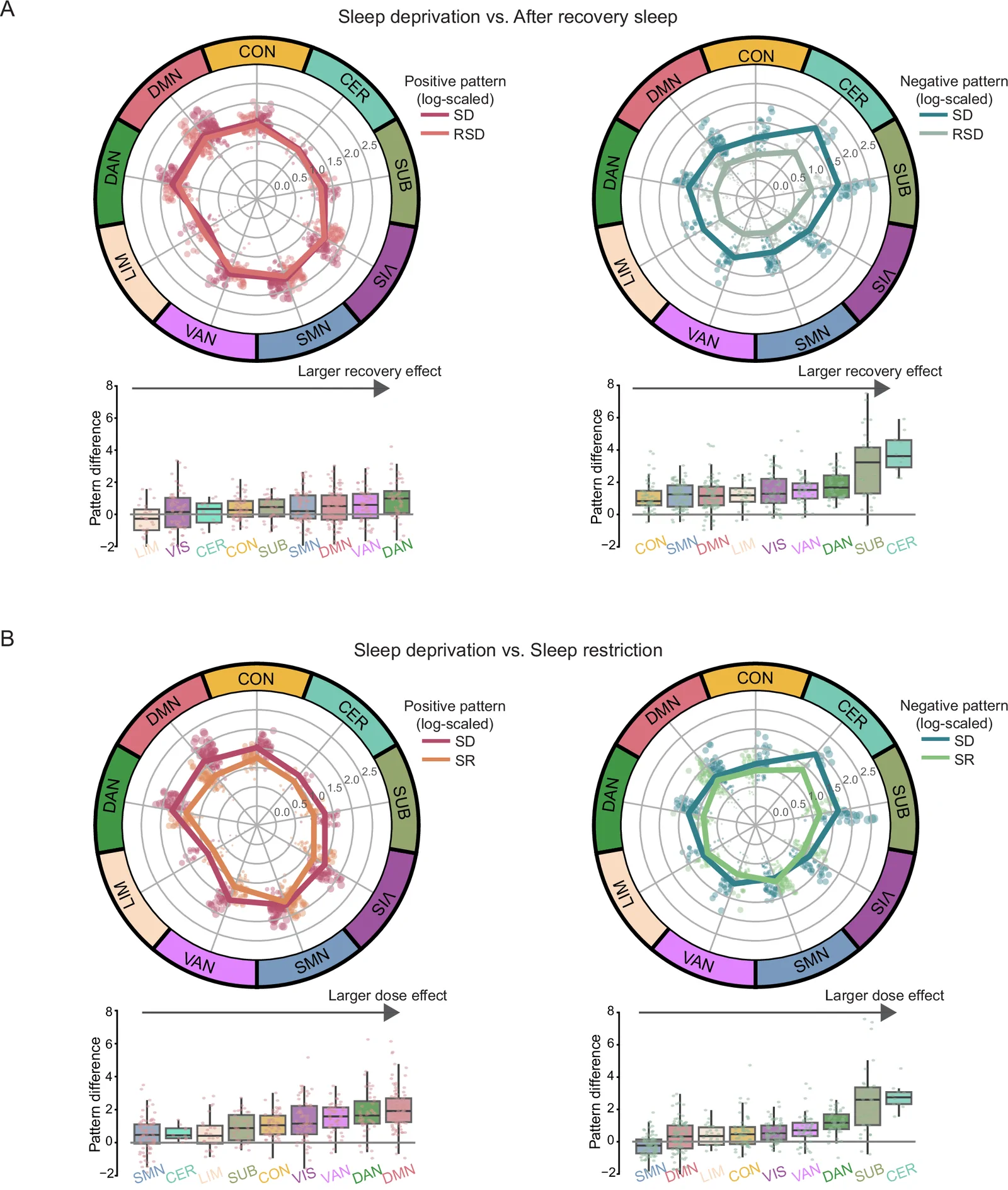
Predictive patterns of different sleep manipulation conditions. **A** Comparing predictive patterns after sleep deprivation and those after recovery sleep. **B** Comparing predictive patterns after sleep deprivation and those after sleep restriction. In the radar plots, each circle represents a brain region, with the size of the circle proportional to its regional predictive pattern amplitude. Positive (left) and negative (right) patterns are shown separately. The values in the radar plots were log-scaled for visualization. In the box plots, the bounds of the box represent the 1st (25%) and 3rd (75%) quartiles, the central line represents the median, and the whiskers represent the values within 1.5 times the interquartile range. Each dot represents a brain region. The number of regions in frontoparietal control (CON), default mode (DMN), dorsal attention (DAN), limbic (LIM), ventral attention (VAN), somatomotor (SMN), visual (VIS), subcortical (SUB), and cerebellum (CER) networks are 52, 91, 46, 26, 47, 77, 61, 32, and 10, respectively. SD sleep deprivation, RSD recovery sleep after sleep deprivation, SR sleep restriction. Source data are provided as a Source Data file.

We further examined how predictive patterns change following a night of recovery sleep. Specifically, we calculated the differences between regional predictive patterns observed after sleep deprivation and those after recovery sleep (see Methods). As shown in Fig. 6A, the differences of the positive predictive patterns between the two conditions were moderate (mean±s.d. regional pattern difference: 0.39 ± 1.08), indicating significant but limited recovery effects. In contrast, the negative predictive patterns exhibited more substantial restoration (1.51 ± 1.22). The overall recovery effect of the positive predictive patterns was significantly smaller than the negative predictive patterns (*t*(440) = -15.8, *p* = 9.8E-45). For the positive patterns, higher-order regions from the DAN (0.89 ± 1.26), VAN (0.53 ± 1.02), and DMN (0.48 ± 1.01) had the largest recovery effects. For the negative patterns, the cerebellum (3.81 ± 1.17) and subcortical network (3.11 ± 2.01) were restored the most compared to the other networks (range from 1.11 to 1.78).

Additionally, we investigated the effects of different doses of sleep manipulation on the predictive patterns, by comparing the patterns observed under sleep deprivation and sleep restriction (Fig. 6B). We found that higher dose of sleep losses led to larger neural impacts on both positive and negative patterns (positive: 1.28 ± 1.23; negative: 0.63 ± 1.26; positive vs. negative: *t*(440) = 8.65, *p* = 9.41E-17). The positive patterns associated with the DMN (2.04 ± 1.25), DAN (1.79 ± 1.35), and VAN (1.50 ± 0.90) were particularly sensitive to the intensity of sleep manipulation, showing notable differences between the two manipulation conditions. For the negative patterns, connections associated with the CER (2.78 ± 0.76) and SUB (2.54 ± 2.07) demonstrated large differences between sleep deprivation and sleep restriction.

## Discussion

We identified a robust and generalizable neural signature of sleep deprivation using machine learning and large neuroimaging datasets. The decoder accurately distinguished sleep-deprived from well-rested individuals, and robustly generalized to independent datasets, underscoring its potential as a reliable biomarker for sleep deprivation. The decoder detected residual neural perturbations in previously sleep-deprived individuals after they underwent a night of recovery sleep. The decoder was also responsive to milder sleep loss and captured natural sleep variability in the general population. The neural signature we identified involves functional connectivity across multiple neural systems, highlighting that sleep deprivation leads to distributed neural perturbations. We also provide insights into distinct neural networks that are vulnerable to sleep manipulations and the heterogeneity of restorative effects after a night of recovery sleep.

The machine learning approaches enabled us to integrate distributed connectivity patterns to construct a neural signature of sleep deprivation and to provide a more holistic picture of its effects on the brain. The neural signature engaged around 10% of whole-brain connections, indicating the broad neural impacts of sleep loss and echoing observations that functional deficits in sleep-deprived brains span various cognitive and emotional tasks. Across neural systems, the default mode (DMN), which is largely implicated in internal mental processes, and the dorsal attention network (DAN), which is normally activated by externally oriented tasks, are among the most important contributors to the positive patterns of the signature. This finding is consistent with prior univariate-based studies^11,12,51,52^, presumably reflecting an impaired balance between internally focused (DMN) and externally directed (DAN) processes, which contributes to deficits in cognitive flexibility and attentional processes after sleep loss.

The cerebellum and subcortical networks were the most significant contributors to the negative predictive patterns. The role of the cerebellum in non-motor functions like learning and memory, emotional processing, and attention is increasingly recognized^53,54^. The cortico-cerebellar connectivity fluctuates across vigilance states, with decreased connectivity observed in low arousal states^55^. Therefore, the reduced cortico-cerebellar connectivity after sleep deprivation may reflect a reduction in arousal level that contributes to functional deficits. The disruption of thalamocortical connectivity after sleep deprivation has been observed in previous studies^9,56^. The thalamus is not only a key contributor for sensory processing and maintaining arousal, but also critical to diverse cognitive and behavioral functions^57^. Recent views consider the thalamus as a connector hub in coordinating communication between large-scale networks^58^. The reduction in thalamocortical connectivity would therefore suggest that sleep deprivation-induced deficits in cognitive functions might be associated with dampened control signals from the thalamus in modulating inter-network communication.

Interestingly, the subcortical regions and the cerebellum, which contributed most to the decoder's negative patterns, showed greater restoration after a single night of recovery sleep than higher-order cognitive networks (e.g., the DMN and DAN). This heterogeneity in recovery effects may reflect the distinct functional demands and homeostatic processes across neural systems. The relatively faster recovery of the subcortical regions (e.g., the thalamus) might be due to its extensive involvement in basic physiological functions, such as arousal regulation^59^, and the rapid recovery of the cerebellum might be due to its necessity in sensorimotor control^60^. A plausible reason for this relatively fast recovery might be that these regions possess greater plasticity or metabolic efficiency^61^, enabling them to recover more rapidly in order to maintain essential physiological processes. In contrast, higher-order cortical networks are responsible for complex cognitive functions^62,63^, such as decision-making and attentional control, which require higher demands of neural integration. These networks may experience more sustained disruption due to their greater metabolic demands and the intricate synchronization needed to support complex behavior. This speculation is aligned with behavioral studies showing persistence of sleep deprivation-induced cognitive deficits after a night of recovery sleep^64,65^. Together, these findings demonstrate that while basic physiological processes recover relatively quickly, higher-order cognitive systems may need more time and prolonged recovery sleep to fully restore their functional integrity.

The sensitivity of this neural signature to partial sleep deprivation highlights the brain’s vulnerability to even moderate reductions in a single night of sleep. The neural effects of acute sleep restriction have been inconsistently reported in the literature^66^. Our results demonstrated the advantages of an integrative machine learning approach in detecting the subtle neural perturbations induced by sleep restriction. It is important to note that the sleep restriction condition in this study was limited to the second half of the night, disproportionately impacting REM sleep. This design choice mirrors real-world situations in which sleep is often shortened toward the end of the night. The similarity in predictive patterns between full sleep deprivation and partial sleep restriction may therefore reflect the shared reduction in REM-rich sleep. At the same time, slow-wave sleep, which predominates in the first half of the night and plays a key role in restorative processes, may have yielded a different neural signature if selectively restricted. Future studies that directly compare the first half versus the second half of night sleep restriction could further elucidate the distinct contributions of REM and slow-wave sleep to the neural consequences of sleep loss.

In real-world scenarios, individuals often experience ongoing continuous partial sleep loss. Prior studies have showed that chronic sleep restriction can accumulate and lead to cognitive impairments similar to those observed in acute sleep deprivation^67–69^, and natural sleep duration associates with functional connectivity^35,70^. Extending these observations, we found that habitual short sleep durations in the general population (from datasets 5 and 6) are associated with higher neural expression resembling that observed after acute sleep deprivation. These results provide strong evidence indicating that even naturally occurring short sleep durations, when sustained over time, may lead to persistent changes in neural connectivity that partially mirror the effects of acute sleep deprivation.

Leveraging the largest sample sizes among sleep deprivation studies to-date, we demonstrated that acute sleep deprivation significantly perturbs brain circuits, enabling robust detection of sleep-deprived individuals. The neural signature we identified provides an objective neurobiological metric for understanding and evaluating the impact of sleep loss. There may be skepticism regarding the utility and necessity of neurobiomarkers for sleep loss–given that there are other easier ways to assess sleepiness, such as subjective self-report of sleepiness, cognitive performance tests, or behavioral assessments (e.g., the multiple sleep latency test or psychomotor vigilance test). However, our findings underscore the distinct advantages of a neural signature. First, subjective measures are susceptible to biases. For example, self-reported and objectively measured sleep duration are only moderately or weekly correlated^71,72^. Second, the effect sizes obtained using the neural signature are comparable to or exceed many cognitive/behavioral tests. When generalized directly to independent datasets (datasets 2 and 3), the neural signature achieved effect sizes (Cohen’s *d*) of 1.90 and 1.35, respectively, which are higher than or at least on par with effect sizes reported for sleep deprivation on self-reported sleepiness (partial eta squared: 0.50 ~ 0.63, converted Cohen’s *d*: 1.02 ~ 1.33)^73^ and cognitive tests (meta-analysis, Hedge’s *g*: 0.13 ~ 0.78, close to Cohen’s *d*)^74^. Finally, and perhaps most importantly, a neural signature offers a systems-level characterization of the distributed neural network alterations associated with sleep deprivation.

The neural signature identified here is best interpreted as a state marker of acute sleep loss, reflecting a composite of sleep-related processes rather than a single psychological construct. Sleep deprivation is known to affect multiple domains, including arousal, alertness, and affect, which are inherently interrelated. Our exploratory movie-watching analysis suggested that the signature may be sensitive to arousal-related fluctuations but not affective valence. Together with the UK Biobank analyses showing specificity to sleep duration rather than other behavioral measures, these findings indicate that the signature is not reducible to general affective states, but instead reflects a sleep-loss–related neural state with some sensitivity to arousal. Future work integrating direct behavioral and physiological measures will be important for further disentangling these components.

The neural signature was developed using task-based fMRI data from a randomized, between-subjects discovery dataset and subsequently generalized to independent resting-state datasets with within-subject sleep-manipulation designs. Functional connectivity estimated during task performance captures coordinated interregional fluctuations and has been shown to preserve large-scale network organization observed during resting-state^75–78^. Moreover, random assignment of healthy participants to sleep conditions in the discovery dataset reduced confounding by pre-existing individual differences. Although dataset 1 did not include resting-state fMRI, the successful generalization of the task-based decoder to independent resting-state datasets suggests that the identified pattern is not specific to the training task or study design, but instead reflects sleep-loss-related connectivity changes that generalize across contexts.

A notable feature of the present study is the heterogeneity across datasets in acquisition protocols, demographic composition, and clinical characteristics. Rather than representing a limitation, this heterogeneity provides a strong test of the neural signature's generalizability. Importantly, the model was trained on a randomized experimental dataset (dataset 1) comprising healthy adults and subsequently evaluated in independent datasets using within-subject cross-over designs (datasets 2–4), enabling the isolation of state-dependent changes associated with sleep manipulation while controlling for stable factors such as age, clinical status, and scanner-related variability. In cross-sectional datasets (datasets 5-6), associations between PoSD and total sleep time were assessed while controlling for potential confounds, including age, sex, head motion, and other cognitive and psychological factors. The consistent performance of the neural signature across these diverse datasets suggests it captures sleep-related changes in neural state that generalize across contexts.

Several limitations of this study should be considered. First, the neural signature was developed based only on acute sleep deprivation in a laboratory setting, which may differ from the impact of chronic sleep disturbances in the real world^79^. Future studies using longitudinal sleep-manipulation designs would be necessary to better understand the neural effects of sustained sleep loss. Second, the decoder was constructed based on a cohort of healthy young adults, and the two validation datasets on sleep deprivation also primarily included young adults. The decoder was successfully generalized to the UK Biobank dataset, which included older adults aged 40 to 69 years without sleep manipulations, suggesting a reasonable degree of generalizability to other cohorts. An important future direction is to examine whether the sleep-deprivation neural signature generalizes to younger populations. Large developmental datasets such as the adolescent brain cognitive development (ABCD) study^80^ could help determine whether PoSD relates to habitual sleep variation earlier in life. Future work leveraging developmental cohorts will be essential for understanding how the neural expression of sleep loss varies across the lifespan. Third, we focused on static functional connectivity, which assumes that the strength of connections is constant over time. However, the interactions between brain regions are dynamically involved during cognitive processing, which may be perturbed by sleep deprivation^81,82^. Combining static and dynamic functional connectivity measures may improve the classification performance and provide additional insights about the effects of sleep disturbances. Fourth, this study was not preregistered, which limits full analytical transparency. However, the primary analyses used a fixed training pipeline applied consistently across multiple independent datasets, including within-subject sleep manipulation samples, reducing analytical flexibility. Future work would benefit from formal preregistration.

Overall, this study provides a robust neural signature that captures the widespread impact of sleep deprivation on whole-brain neural networks. The sensitivity of this signature to both full and partial sleep deprivation, as well as its association with natural sleep variability in individuals not undergoing any sleep manipulations, underscores its potential as a biomarker for sleep loss. The persistence of disturbances in neural networks, despite recovery sleep, emphasizes the complexity of sleep recovery and the need for further research into these dynamic processes.

## Methods

This study complied with all relevant ethical regulations. All participants provided written informed consent prior to participation. The study protocols for each dataset were approved by the corresponding institutional review boards or research ethics committees at the institutions where data collection was conducted. Sex was self-reported by participants. Sex/gender were not a primary focus of this study; thus, no sex- or gender-based analyses were conducted. Details of each dataset are provided below.

### Discovery dataset (dataset 1)

#### Participants and experimental procedure

This dataset included 151 healthy adults ages 18–30 years (mean ± s.d.: 23.8 ± 3.3; 69 male, 82 female). All participants were free of current sleep, psychiatric or medical disorders. The study was approved by the University of Pittsburgh Institutional Review Board and the Department of Defense Human Research Protection Office. All participants provided informed consent before attending the study and received financial compensation for their participation.

#### Experimental design

Before the experimental phase of the study, participants were asked to complete a sleep diary and wear an actigraph for 7 to 10 consecutive nights and days to ascertain that they maintained regular sleep/wake schedules and obtained sufficient sleep. Only participants who demonstrated actigraphy-based regular rise times between 05:30 and 8:30, and regular bedtimes between 22:00 and 01:00, and who showed a habitual sleep duration between 6 and 9 h, and sleep efficiency > 80% for the 10 days preceding the laboratory experiment were invited to complete the laboratory study.

Participants underwent three experimental days in the laboratory. On the night of day 1, participants were randomized to one of three sleep conditions: normal sleep (NS), sleep restriction (SR), or sleep deprivation (SD). Participants in the NS group went to sleep at their habitual bedtime and rose at their habitual rise time. Participants in the SR group were awakened after 50% of their habitual sleep and remained awake during the second half of the night. Participants in the SD group remained awake throughout night 1. On day 2, participants underwent two MRI sessions, one at morning (9 am to 10 am) and the other at evening (6 pm to 8 pm). At the morning MRI session, participants underwent two experimental phases of a threat conditioning paradigm: a threat conditioning phase (13 min.) and an extinction learning phase (13 min.). At the evening session, participants underwent an extinction memory recall phase (13 min.) of the threat conditioning paradigm and finished a reward-related task (8 min.) and a face perception task (11 min.). Participants remained awake throughout day 2 until their bedtime. All participants then had a recovery sleep at their habitual bedtime and rose at their habitual rise time. On the morning of day 3, participants underwent an MRI session, during which they finished a renewal phase (13 min.) of the threat conditioning paradigm and underwent a reward-related task (8 min.) and a face perception task (11 min.) again. After that, participants were discharged.

#### Neuroimaging data acquisition and preprocessing

Neuroimaging data of all participants were obtained using a 3.0 T Siemens Trio MRI scanner. High-resolution T1-weighted (T1w) structural images were acquired using a magnetization-prepared rapid gradient echo (MPRAGE) sequence (slice number = 192, repetition time [TR] = 2200 ms, echo time [TE] = 3.31 ms, flip angle = 9°, field of view [FOV] = 256 × 192 mm^2^, voxel size = 0.9 × 0.9 × 1.0 mm^3^). Functional MRI images were collected using a gradient echo-planar imaging (EPI) sequence (slice number = 39, TR = 2000 ms, TE = 28 ms, flip angle = 90°, FOV = 20.5 mm^2^, matrix = 64 × 64, thickness = 3.1 mm). All three scanning sessions used the same parameters. An eye-tracking system was used to monitor the participants to ensure that the participant remained awake during the tasks.

Imaging data were preprocessed using fMRIPrep 20.0.2. The T1w images were corrected for intensity non-uniformity with N4BiasFieldCorrection (ANTs 2.3.3) and used as T1w-reference throughout the preprocessing. The T1w-reference was skull-stripped, segmented into cerebrospinal fluid, white-matter and gray-matter, and then spatially normalized into the Montreal Neurological Institute (MNI) space (MNI152NLin2009cAsym) through nonlinear registration with antsRegistration (ANTs 2.3.3). The functional images were head-motion-corrected using mcflirt (FSL) and slice-timing-corrected using 3dTshift (AFNI). The preprocessed functional images were then co-registered to the T1w-reference using flirt (FSL) with the boundary-based registration (nine degrees of freedom), and spatially normalized into the MNI152NLin2009cAsym space by applying the parameters obtained from T1w-reference spatial normalization. Normalized functional images were resampled to 2 × 2 × 2 mm voxel size using Lanczos interpolation (ANTs 2.3.3) and smoothed with a 6-mm full-width half-maximum (FWHM) Gaussian kernel.

Brain volumes with framewise displacement (FD) larger than 0.3 mm were censored to reduce the impacts of high-motion volumes. Functional runs with mean FD larger than 0.5 were removed for further analysis. The following nuisance covariates, including six head motion parameters, averaged white matter signal, averaged cerebral spinal fluid (CSF) signal, their temporal derivatives, quadratic terms, and squares of derivatives, were regressed out from the brain signals. The global signals were also regressed out following previous sleep deprivation studies^13,40^. Similar performance was obtained by removing global signal regression from the fMRI preprocessing workflow (Supplementary Fig. 1). The brain time series were band-pass (between 0.01 and 0.08 Hz) filtered to reduce low-frequency drift and high-frequency noise.

#### Functional connectivity estimation

We used a whole-brain parcellation (442 regions in total) comprising 400 cortical regions^37^, 32 subcortical regions^38^, and 10 cerebellum regions^39^. For each fMRI run, time series of the 442 brain regions were extracted. To increase the reliability of FC^75,76^, time series of all fMRI runs from the same session (e.g., morning after sleep manipulation) were concatenated for FC estimation. On average, there were 694 ± 102, 884 ± 98, and 876 ± 102 time points for the morning session after sleep manipulation, the evening session after sleep manipulation, and the morning session after recovery sleep, respectively. We ignored the task structures and used all available runs to estimate a single functional connectivity (FC) matrix for each session^75,83^. We did not regress out task regressors since task regression may reduce reliability of estimated FC^75^. Previous studies have demonstrated that FC matrixes estimated task-based and resting-state FC are highly similar^77,78^, suggesting that key intrinsic connectivity patterns remain consistent across different states. Therefore, our results are unlikely to be specific to the experimental tasks that the participants performed. The high generalization performance on dataset 2 (resting-state) and dataset 3 (resting-state and tasks different from dataset 1) further confirmed that the identified signature is not dependent on specific task states. The Pearson’s correlation between the time series of each pair of regions were calculated, yielding a 442 × 442 FC matrix for each participant and each experimental session. The FC matrix was z-scored using Fisher’s r-to-z transformation, and the upper triangular portion of the FC matrix (a total of 97,461 connections) were used as features for classification analyses.

### Dataset 2

#### Participants and experimental procedure

This dataset is from Chai et al., including resting-state fMRI data from 68 participants^40^, including 30 participants with Major Depression Disorder (age: 34 ± 9.9 years; 7 male, 23 female) and 38 healthy controls (age: 33.5 ± 8.6 years; 21 male, 17 female). All participants have data from two sessions of MRI available, one session before and the other session after a night of sleep deprivation (pre-SD and post-SD sessions). After the participants had a night of normal sleep, they underwent the pre-SD session in the morning of day 1 (7 am to 10 am). After that, the participants were kept awake throughout day 1 and thus were sleep-deprived on the night of day 1. At the morning of day 2 (8 am to 10 am), participants underwent the post-SD session. The resting-state run for each scanning session lasted 7 min. A MR-comparable video camera was used to monitor participants’ eyes to confirm that they did not fall asleep in the scanner. The study was approved by the Institutional Review Board of the University of Pennsylvania and was carried out in accordance with the Declaration of Helsinki. All participants provided written informed consent prior to participation and received financial compensation.

#### Neuroimaging data acquisition and preprocessing

All image data were collected using a 3 T Siemens Trio system with an eight-channel head coil. A multiband gradient-echo EPI sequence was used for resting-state data collection (TR = 2000 ms, TE = 24 ms, flip angle = 90°, matrix = 64 × 64 × 36, slice thickness = 4 mm). A high-resolution (0.8 × 0.8 × 0.8 mm) T1w anatomical scan was collected after the resting-state run. The preprocessing was performed by authors of the original study^40^ using the Statistical Parametric Mapping software (SPM 12, Wellcome Department of Cognitive Neurology, UK) and the DPABI V6.2 toolbox. The performed preprocessing procedures mainly included: 1) motion correction and slice timing correction; 2) removal of nuisance covariates, including nine confounding signals (six head motion parameters, averaged global signal, averaged white matter signal, averaged CSF signal), and their temporal derivative, quadratic term, and temporal derivatives of each quadratic term (24 regressors total); 3) spatial normalization to the MNI space; 4) removal of linear trends and band-pass filtering (0.01–0.08 Hz); 5) spatial smoothness using a 8 mm FWHM Gaussian kernel. The preprocessed data were used to estimate functional connectivity using the same procedure as for the discovery data.

### Dataset 3

#### Participants and experimental procedure

This dataset is from the Perturbation of the Depression Connectome (PDC) study^42^. The PDC study includes a large number of patients with treatment-resistant depression (TRD) who received one of three fast-acting interventions: electroconvulsive therapy, ketamine infusion, or total sleep deprivation, and also healthy controls (HC). Here, we only focused on 54 participants (TRD: *n* = 44; HC: *n* = 10; age: 30.6 ± 9.4 years; 23 male, 31 female) whose preprocessed resting-state fMRI data from pre- and post-sleep-deprivation sessions were available in the PDC 1.0 data release. These participants underwent a pre-SD and a post-SD MRI session. Participants were visually monitored throughout the scan. Multiple data modalities were collected, while we focused on fMRI data in this study. All participants provided written informed consent prior to participation and received financial compensation for their participation. The study protocol was approved by the University of California, Los Angeles institutional review board.

#### Neuroimaging data acquisition and preprocessing

Neuroimaging data were collected using a Siemens 3 T Prisma with a 32-channel head coil. High-resolution T1w structural images were acquired using a multi-echo MPRAGE sequence (slice number = 208, TR = 2500 ms, TE = 1.8/3.6/5.4/7.2 ms, flip angle = 8°, FOV = 256 × 240 mm^2^, voxel size = 0.8 × 0.8 × 0.8 mm^3^). Functional MRI images were collected using a multiband gradient-echo EPI sequence (multiband factor = 8, slice number = 72, TR = 800 ms, TE = 37 ms, flip angle = 52°, FOV = 208 × 208 mm^2^, matrix = 104 × 104, thickness = 2 mm). The detailed scanning protocol was described elsewhere^84^. Functional data were collected while participants underwent resting-state and also multiple tasks. Preprocessing was conducted by investigators of the PDC study and followed the Human Connectome Project (HCP) fMRIVolume pipeline^85^. We estimated FC matrix for each participants using the time series concatenated across all functional runs (both resting-state and tasks, 23 min in total) from each session.

### Dataset 4

#### Participants and experimental procedure

This dataset is from the Stockholm sleepy brain study^25,43^, including 78 healthy adults who participated in a sleep restriction (3 h sleep) experiment. The participants were either younger (20–30 years; 21 male, 21 female) or older (65–75 years; 17 male, 19 female) healthy volunteers. The participants underwent two MRI sessions in two different visits to the laboratory. One session was performed in the evening following normal sleep, the other session was performed in the evening following sleep restriction. The order of the two sessions were counter-balanced across participants. There were two resting-state runs (each lasted 8 min) for each MRI session, separated by an hour of other experimental tasks. During scanning, participants were monitored by eye-tracking. In case of eye-closures of more than 5 s, the MRI operator spoke a wake-up call through the participant’s headphones. Since fMRI data collected during other experimental tasks were optimized for detecting task-related activations thus did not cover the whole brain, we only used data from resting-state runs. All participants provided written informed consent prior to participation and received financial compensation for their participation. The study was approved by the Regional Ethics Review board of Stockholm.

#### Neuroimaging data acquisition and preprocessing

Neuroimaging data were collected using a General Electric Discovery 3 T MRI scanner. Functional images were acquired using the following settings: TR = 2500 ms, TE = 30 ms, flip angle = 75°, slice thickness = 3 mm, slice number = 49, field of view = 28.8 cm. T1w images were acquired with a sagittal BRAVO sequence (field of view = 24 cm, slice thickness = 1 mm). We used the same fMRI data preprocessing workflow as for the discovery dataset. The two resting-state runs from each session were concatenated for FC estimation.

### Dataset 5

#### Participants and experimental procedure

This dataset is from a study previously published from our group^44^. Participants with neuroimaging and actigraphy data available were included in the analysis (*n* = 109; age: 24.7 ± 5.0 years; 40 male, 69 female). All participants underwent a two-day threat-conditioning paradigm in the scanner, including a threat-conditioning phase and an extinction-learning phase on day 1, and an extinction-memory phase on day 2. We used the data from day 1 only, because day 1 contained longer fMRI data acquisition (two phases, 13 min. per phase), which benefited stable FC estimation.

Before their MRI sessions, participants completed an approximately 2-week sleep-assessment period (mean 15.03 ± 3.4 days) during which they continuously wore an Actiwatch-2 (Phillips Respironics, Bend, OR). Participants marked their time-in-bed daily via the Actiwatch-2. The default algorithm of Actiware 5.61 scored 1-min epochs within time-in-bed as sleep or wake and calculated objective values for total sleep time. All participants provided written informed consent prior to their participation and received financial compensation for their participation. All study procedures were approved by the Partners Healthcare Institutional Review Board.

#### Neuroimaging data acquisition and preprocessing

Neuroimaging data were collected using a 3.0 T Siemens Prisma MAGNETOM scanner. High-resolution T1-weighted (T1w) structural images were acquired using a MPRAGE sequence. Functional MRI images were collected using a gradient echo-planar imaging sequence (slice number = 48, TR = 2560 ms, TE = 30 ms, flip angle = 90°, voxel size = 3 × 3 × 3 mm). We used the same fMRI data preprocessing workflow as for the discovery dataset. The conditioning and extinction phases from each participant were concatenated for FC estimation.

### Dataset 6

#### Participants and experimental procedure

This dataset was obtained from UK Biobank^45^, a population-based prospective cohort study. We analyzed a total of 39,799 adults (age: 63.9 ± 7.7 years; 18,623 male, 21,176 female) whose preprocessed fMRI resting-state regional time series^46^ and subjective reported habitual sleep duration (data field: 1160) were available. For a subset of participants, their accelerometry-derived sleep durations were also available (resource returned to UK Biobank by Walmsley and colleagues^47^, data field: 40046). The accelerometry data were collected from these participants who worn an Axivity AX3 wrist- triaxial accelerometer for 7 days. These data were fed to a machine learning method to estimate sleep duration (range from 0 to 1), which was defined as the overall average proportion of time spent sleeping across the monitoring period (see^47^ for details). Participants with unusual measures were excluded (criterion: estimated sleep duration less than 0.17 or more than 0.59, roughly less than 4 h or more than 14 h), which resulted in 17,209 participants with both fMRI data and accelerometry-derived sleep durations available. All participants provided written informed consent prior to their participation. Participants were reimbursed for their travel expenses in accordance with study procedures. The protocol of UK Biobank was approved by the North West Multi-center Research Ethics Committee. The use of UK Biobank data is governed under the Approved Research Project #34077.

#### Neuroimaging data acquisition and preprocessing

Resting-state fMRI data were collected on 3T Siemens Skyra scanners using a 32-channel head coil. The T1-w structural images were acquired using a MPRAGE sequence. Resting-state fMRI data were acquired with a multi-band gradient echo EPI sequence. The resting-state run included 490 volumes (around 6 min) using the following parameters: multi-band = 8, TR = 735 ms, TE = 39 ms, flip angle = 52°, voxel size = 2.4 × 2.4 × 2.4 mm. Since preprocessing for this large sample is quite time-consuming, we used a resource returned to UK Biobank by Mansour and colleagues^46^, which contained preprocessed regional time series of the 400 cortical regions and the 32 subcortical regions. The preprocessing workflow followed standard procedure for resting-state data processing and was detailed elsewhere. We estimated the FC matrix for each participant using the downloaded time series with the mean global signal regressed out. Note that the shared data did not include time series of the ten cerebellum regions. Thus, we retrained a classifier using the discovery dataset with the ten cerebellum regions excluded, and applied this classifier for UK Biobank data.

### Classification analysis

#### Neural signature construction using the discovery dataset

We developed a whole-brain FC-based neural signature predictive of sleep-deprived brains using data from the discovery dataset (dataset 1). We used FC measures from the morning MRI session after sleep deprivation as features to classify SD (*n* = 52) vs. NS (*n* = 48). We employed support vector machine (SVM) with linear kernel from scikit-learn as the classifier. We assessed the classification performance on the discovery dataset using a 10 × 10 cross-validation procedure. Specifically, we evenly divided participants from the discovery dataset into ten folds, trained the classifier using data from nine folds of the participants, and tested the performance using data from the left-out participants. This procedure was repeated ten times, with each fold of participants being the test set once. To avoid a potential bias of dataset split, we repeated the cross-validation procedure ten times, using different splits in each repetition. Mean performance, i.e., the area under the receiver operating characteristic curve (AUC), across all ten repetitions was used as the final performance.

To further investigate the classification performance across MRI sessions, we conducted an internal dataset validation using data from another session (the evening after sleep deprivation) as a test set. Specifically, we evenly divided participants into ten folds. We trained the classifier using data from nine folds of the participants’ morning session, and tested the performance using data from the left-out participants’ evening session (instead of the morning session). In this way, the training and testing sets were from different participants and different sessions.

Permutation tests were used to determine the statistical significance of the classification performance. Specifically, we randomly permuted the labels of the participants 1000 times. We conducted the cross-validation procedure as described above using the permuted labels to obtain the null distribution of the classification accuracy. The *p*-value was calculated by counting the percentage of accuracies within the null distribution that was higher than the accuracy obtained with the real data.

#### Generalization of the neural signature on independent datasets

The external generalizability of the classifier on identifying sleep-deprived brains was examined using datasets 2 and 3. We constructed the neural signature (classifier) using all data from the discovery dataset and applied it to two datasets that were never used during training to evaluate classification performance. The two FC feature vectors of each participant (one from pre-SD and the other from post-SD) were fed to the classifier to obtain two scores of the probability of being sleep-deprived (PoSD). The PoSD was calculated using a Platt scaling procedure to convert the SVM classifier's decision values into probabilities. Briefly, the Platt scaling procedure fitted a logistic regression model to the decision values (based on training data), thereby transforming them into calibrated probabilities bounded between 0 and 1. A forced-choice test was used to evaluate the classification performance on each dataset. In the forced-choice test, the feature vector that led to a higher PoSD was classified as post-SD, and the other was classified as pre-SD. The forced-choice accuracies were averaged across participants. Two-sided binomial tests were used to evaluate the significance of the forced-choice accuracies.

#### The neural signature under other sleep conditions

We first examined whether a recovery sleep could restore sleep-deprived brains to a normal level. We conducted the analysis on the discovery dataset, which included a recovery sleep night for all participants. We evenly divided participants (SD and NS groups) from dataset 1 into ten folds. We trained the classifier using data from nine folds of the participants’ morning session after sleep deprivation, and tested the performance using data from the left-out participants’ morning session after recovery sleep. If sleep-deprived brains were fully restored after recovery sleep, the classification performance would be at chance level.

We also examined the expression of the SD neural signature in participants who were sleep-restricted (i.e., partially sleep-deprived). This analysis was conducted on two different datasets. The first dataset included two MRI sessions from the SR group of the discovery dataset (*n* = 51): one at the morning after sleep restriction, the other at the morning after these individuals underwent a recovery sleep. The second dataset included two MRI sessions from dataset 4 (*n* = 76): one after normal sleep, and the other after sleep restriction. PoSD scores were obtained for each session and participant and compared between sessions.

#### The neural signature correlates with habitual sleep duration

The correlation between PoSD and habitual sleep duration was examined on datasets 5 and 6. For dataset 5, the habitual sleep duration of each participant was estimated using actigraphy data collected before the MRI session. The signature expression (PoSD score) was obtained by applying the constructed classifier to each participant’s fMRI data, and used partial correlation to correlate PoSD with the sleep duration, controlled for the scanning site, movement during scanning, age and sex. For dataset 6, the habitual sleep duration of each participant was obtained using the participants’ reported hours of regular sleep. Participants were stratified to groups based on their sleep hours. Since a small number of participants reported sleep hours less than 6 h or more than 9 h, these participants were assigned to a group with <6 h of sleep, and a group with >9 h of sleep, respectively. In total, there were six sleep groups: <6 h, 6 h, 7 h, 8 h, 9 h, >9 h. An analysis of covariance (ANCOVA) was used to examine the difference of PoSD across groups: the PoSD was the dependent variable, the sleep group was the independent variable, the scanning site, movement during scanning, age and sex were included as covariates. For participants with accelerometry-derived sleep durations available (n = 17,209), a partial correlation was calculated between PoSD and accelerometry-derived sleep duration, with the covariates mentioned above controlled for.

#### Predictive patterns contributed to the classification

We examined the contribution of each connection for identifying sleep-deprived brains. We first extracted the model weights from the classifier trained using the discovery dataset. We then used the Haufe transformation^50^ to convert model weights into predictive patterns, which are more reliable than model weights for representing the contributions of connections to classification. The predictive pattern provides an intuitive interpretation of the contribution of each connection in the predictive model: a positive predictive weight indicates that this connection increases after SD, while a negative predictive weight indicates that it decreases after SD. We further conducted a permutation test to identify connections with significant predictive weights. Specifically, we randomly shuffled the labels of the discovery dataset and re-estimated the predictive patterns 1000 times, which generated the null distribution of predictive weight for each connection. We then calculated z-scores and two-tailed *p*-values based on the mean and standard deviation of the null distribution. The significant connections were identified based on false discovery rate (FDR) corrected *p*-values (*p*  <  0.05, two-sided). To examine the contributions of different neural systems, we divided the brain regions into nine intrinsic neural subnetworks^37–39^: the frontoparietal control network (CON), default mode network (DMN), ventral attention network (VAN), dorsal attention network (DAN), somatomotor network (SMN), visual network (VIS), limbic network (LIM), subcortical network (SUB), and cerebellum network (CEB). We calculated the number of significant connections within each subnetwork and between every two subnetworks. Since different subnetworks included different numbers of regions, we also calculated the percentages of significant connections within or between subnetworks among all possible connections. The regional positive/negative patterns were calculated by summing all positive/negative patterns associated with each region. For the negative patterns, the absolute values were used in the analysis.

#### Comparing predictive patterns across experimental conditions

We compared the predictive patterns of sleep deprivation with those after recovery sleep and after sleep restriction. These analyses were based on the discovery dataset. The predictive pattern after a recovery sleep was estimated by training a classifier to classify SD vs. NS using data from the morning session after recovery sleep (dataset 1). The predictive pattern of sleep restriction was estimated by training a classifier to classify SR vs. NS using data from the morning session after sleep restriction (dataset 1). The classifier weights were transformed to predicative patterns using Haufe transformation^50^. We then calculated the regional positive/negative patterns and compared them to the regional patterns of the identified neural signature.

### Reporting summary

Further information on research design is available in the Nature Portfolio Reporting Summary linked to this article.

## Supplementary information

Supplementary Information

Reporting Summary

Transparent Peer Review file

## Source data

Source Data

## Data Availability

The processed data of Dataset 1 are available at https://osf.io/ayj72/. The raw data are not publicly available due to a lack of current institutional authorizations for public data sharing. The raw data could be available upon request and engage in a data-sharing agreement with the institution. Requests for Dataset 1 should be directed to A.G. (anne.germain.phd@gmail.com). Dataset 2 is available at https://osf.io/ubhpx/. Dataset 3 is from the Perturbation of the Depression Connectome (PDC) 1.0 data release at https://www.humanconnectome.org/. Dataset 4 is available in OpenNeuro database with accession number ds000201. Dataset 5 is available at NIMH Data Archive through collection ID 2393. Dataset 6 is available from UK Biobank, which can be accessed by applying for access via its Access Management System at https://www.ukbiobank.ac.uk/enable-your-research/apply-for-access. Source data are provided with this paper.

## Code availability

Custom codes to generate the main results are available on GitHub at https://github.com/zhenfu-wen01/sleep_loss_signature and in the Zenodo repository^86^ (https://zenodo.org/records/20822243).
