# Supplementary material for: A neural signature of sleep deprivation in the human brain
Source: Nat Commun. 2026 Jul 22;17:8919. doi: 10.1038/s41467-026-75661-x (PMC13503868; doi:10.1038/s41467-026-75661-x)
Supplement: Supplementary file 1 [file 41467_2026_75661_MOESM1_ESM.pdf]

Supplementary Information

A neural signature of sleep deprivation in the human brain

Zhenfu Wen, Edward F. Pace-Schott, Peter L. Franzen, Lihan Cui, Kai Zhang, Si Gao, L. Elliot Hong, Peter Kochunov, Anne Germain, Mohammed R. Milad

Results

Multivariate pattern analysis is more robust and sensitive than univariate analysis

To evaluate the robustness and sensitivity of multivariate pattern analysis (MVPA) compared to univariate analysis, we conducted univariate analysis to compare functional connectivity between sleep-deprived and well-rested brains. For dataset 1, we used a two-sample t-test to compare the SD group and NS group based on the morning fMRI session after sleep manipulation. For datasets 2-3, we performed paired t-tests to compare the sleep-deprived and well-rested states of the same participants. Following multiple comparison correction (false discovery rate [FDR], $p < 0.05$, **Supplementary Fig. 2A**), we identified 7,134 significant connections in dataset 1; 14,946 in dataset 2; and 30,170 in dataset 3. However, only a small subset of connections (n = 1,972) was consistently identified as significant across all three datasets, representing 27.6%, 13.2%, and 6.5% of significant connections in datasets 1-3, respectively. This low consistency across datasets suggests limited reproducibility of univariate analysis.

We further calculated effect sizes (Cohen's d) for each connection within each dataset. The effect size distributions were similar across the three datasets (**Supplementary Fig. 2B**), with the top 1% largest effect sizes (absolute values) around 0.9 (0.88, 0.80, and 0.94 for datasets 1-3, respectively). The largest effect sizes across all datasets did not exceed 1.45 (1.44, 1.32, and 1.38 for datasets 1-3, respectively). To directly compare the sensitivity of univariate and multivariate analyses, we converted the area under the curve (AUC) obtained from dataset 1 (**Fig. 1B**) into Cohen's d, and estimated effect sizes (sleep-deprived vs. well-rested states) based on the probability of being sleep-deprived (PoSD) scores on datasets 2-3 (**Figs. 1C**&**1D**). The effect sizes derived from MVPA were 1.90, 1.90, and 1.35 for datasets 1-3, respectively, which were higher or at least comparable to the largest effect sizes identified through univariate analysis. Overall, these results suggested that MVPA is more robust and sensitive than univariate analysis in identifying neural alterations associated with sleep deprivation. This advantage is particularly notable considering that MVPA provides out-of-sample prediction for each individual.

Association between the probability of being sleep-deprived and other measures in the UK Biobank dataset

To evaluate the specificity of the neural signature, we examined the associations between the probability of being sleep-deprived (PoSD) and a range of behavioral and psychological measures in the UK Biobank cohort. Measures were defined following our prior work using the UK Biobank dataset[1]. Cognitive measures were derived using tasks from the UK Biobank cognitive test battery, including reaction time, numeric memory, symbol digit substitution, matrix reasoning, tower rearranging, and prospective memory, which capture domains such as processing speed, working memory, reasoning, executive function, and fluid intelligence. Additional measures included fluid intelligence, physical activity (derived from the International Physical Activity Questionnaire-Short form), nicotine use (average number of pack-years smoked by an individual each year over adulthood), alcohol intake (by multiplying frequency of drinking alcohol and amount of alcohol drunk on a typical drinking day), cannabis use (total times of cannabis use in their

lifetime), and adverse childhood experiences (based on questionnaire items adapted from the Childhood Trauma Screener).

For each participant, PoSD values were obtained by applying the trained decoder to resting-state functional connectivity data. Associations between PoSD and each measure were quantified using partial correlations, controlling for age, sex, scanning site, and head motion. Results are summarized alongside correlations between PoSD and self-reported and accelerometer-based total sleep time in **Supplementary Table S1**. To further assess specificity, we conducted additional partial correlation analyses to test whether associations between PoSD and total sleep time remained significant when all other measures were included as covariates. These analyses yielded similar results as in our main analysis (self-reported: r = -0.067, p = 1.05E-40; accelerometer-based: r = -0.096, p = 1.59E-36).

Association between the probability of being sleep-deprived and arousal/valence measures

To provide initial insight into the psychological processes reflected by the neural signature, we conducted an exploratory analysis using the Friday Night Lights (FNL) dataset (Chang et al., 2021[2]; n = 35). In this dataset, participants underwent fMRI while watching a movie, and continuous arousal and valence ratings were obtained from a separate group of participants (Ke et al., 2025[3]).

For each participant, the continuous arousal and valence time series were standardized and then averaged across participants to generate group-level arousal and valence ratings. For each dimension separately, time points were divided into high and low segments using a median split, with time points above the median assigned to the high condition and those below the median assigned to the low condition. Whole-brain functional connectivity matrices were then estimated separately for the high and low segments for each participant. The trained sleep-deprivation decoder derived from Dataset 1 was then applied to each connectivity matrix to obtain probability of sleep deprivation (PoSD) scores for the high- and low-arousal segments and for the high- and low-valence segments.

To test whether PoSD differed between conditions, we fitted linear mixed-effects models with condition (high vs. low arousal, or high vs. low valence) and framewise displacement (FD) as fixed effects and participant as a random intercept. After controlling for FD, PoSD scores were significantly higher during high-arousal than low-arousal segments, suggesting that the neural signature was more strongly expressed during moments of elevated arousal (b = 0.074, z = 3.244, p = 0.001, 95%CI = [0.029, 0.119]). By contrast, no significant difference in PoSD was observed between high- and low-valence segments (b = -0.016, z = -1.079, p = 0.281, 95%CI = [-0.044, 0.013]).

References

1. Ma, Y. *et al.* Association between brain similarity to severe mental illnesses and comorbid cerebral, physical, and cognitive impairments. *NeuroImage* 265, 119786 (2023).
2. Chang, L. J. *et al.* Endogenous variation in ventromedial prefrontal cortex state dynamics during naturalistic viewing reflects affective experience. *Sci. Adv.* 7, eabf7129 (2021).
3. Ke, J., Song, H., Bai, Z., Rosenberg, M. D. & Leong, Y. C. Dynamic brain connectivity predicts emotional arousal during naturalistic movie-watching. *PLOS Comput. Biol.* 21, e1012994 (2025).

Supplementary Table S1. Association between the probability of being sleep-deprived and different measures.

Self-reported total sleep time	Accelerometer-based total sleep time	Matrix	Reaction time	Numeric memory	Symbol digit	Tower
r = -0.067, *p* = 5.28E-41	*r* = -0.096, *p* = 3.96E-36	*r* = 0.020, *p* = 9.27e-4	*r* = -0.012, *p* = 0.021	*r* = 0.012, *p* = 0.054	*r* = 0.026, *p* = 1.60E-5	*r* = 0.014, *p* = 0.021
**Prospective memory**	**Fluid intelligence**	**Adverse childhood experience**	**Physical activity**	**Nicotine use**	**Cannabis use**	**Alcohol intake**
r = 0.005, *p* = 0.356	*r* = -0.013, *p* = 0.015	*r* = 0.007, *p* = 0.225	*r* = 0.008, *p* = 0.149	*r* = -0.005, *p* = 0.645	*r* = 0.006, *p* = 0.314	*r* = -0.040, *p* = 2.80E-11

All statistical tests were two-sided. No adjustments were made for multiple comparisons.

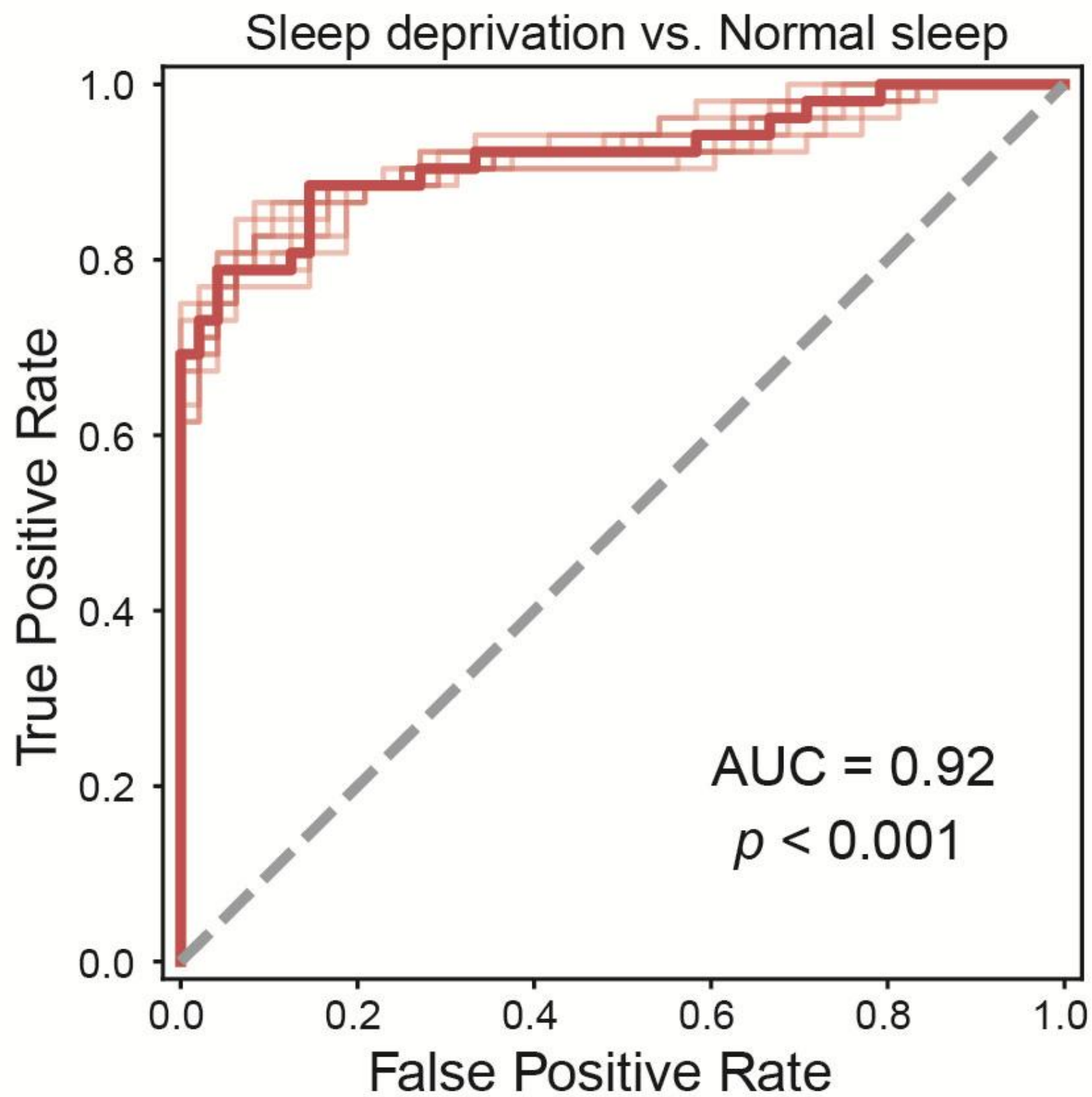

Supplementary Fig. 1. Cross validation performance without conducting global signal regression. With (**Fig. 1B** in the main text) or without global signal regression in neuroimaging preprocessing workflow led to similar classification performance (sleep deprivation vs. normal sleep, morning after sleep manipulation; $n = 100$). AUC: area under the receiver operating characteristic curve. Source data are provided as a Source Data file.

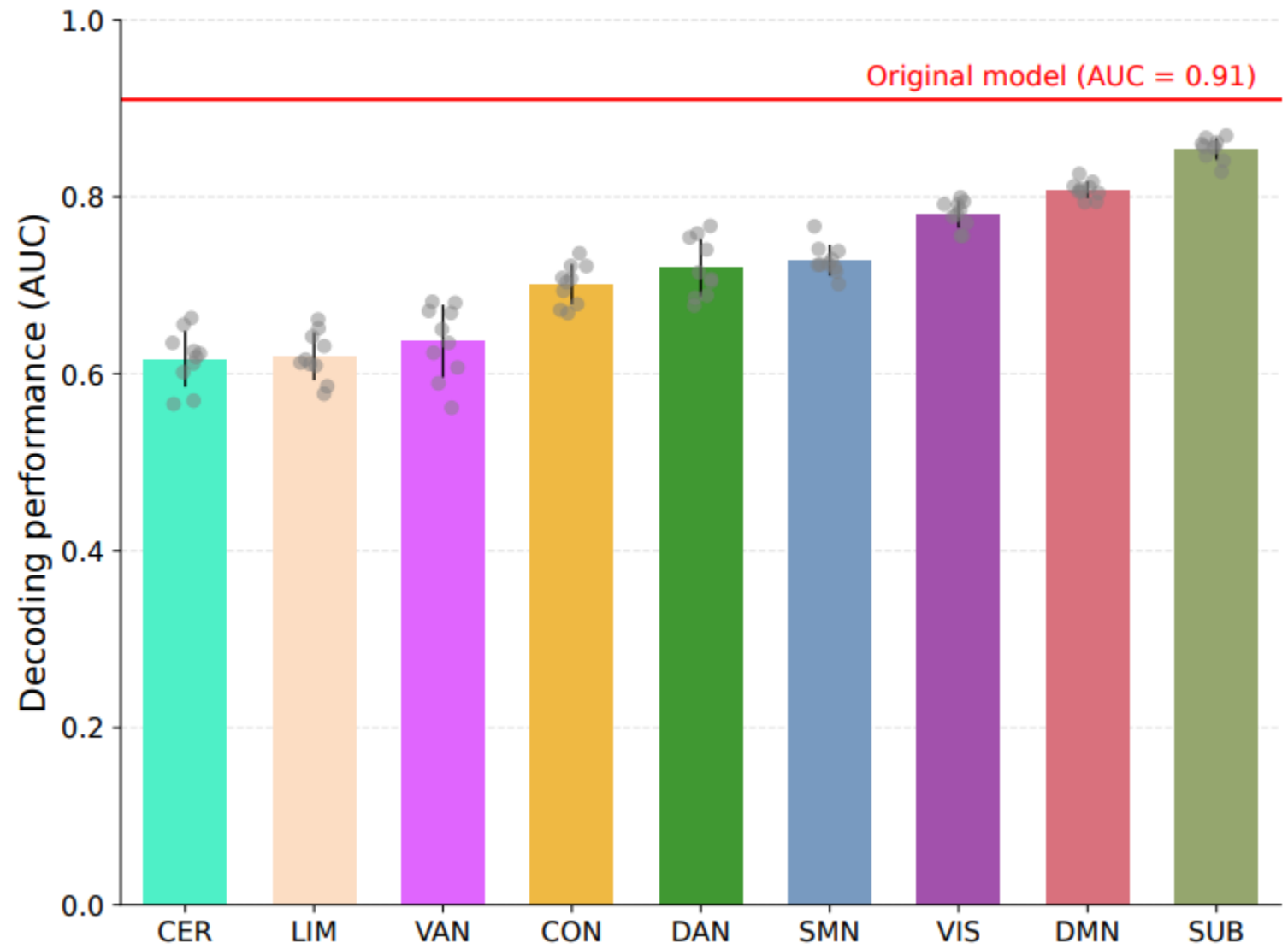

Supplementary Fig. 2. Classification analysis based on functional connectivity within individual canonical networks. To evaluate whether the predictive performance of the neural signature depends on distributed connectivity patterns, we conducted additional analyses comparing the whole-brain model with models based on reduced feature sets. Specifically, for each canonical network, we constructed classifiers using only within-network functional connections. While some individual networks achieved relatively high classification performance, none matched the performance of the whole-brain model (*n* = 100). Error bar represents the standard error of the mean. AUC: area under the receiver operating characteristic curve. CON: frontoparietal control network; DMN: default mode network; VAN: ventral attention network; DAN: dorsal attention network; SMN: somatomotor network; VIS: visual network; LIM: limbic network; SUB: subcortical network; CEB: cerebellum network. Source data are provided as a Source Data file.

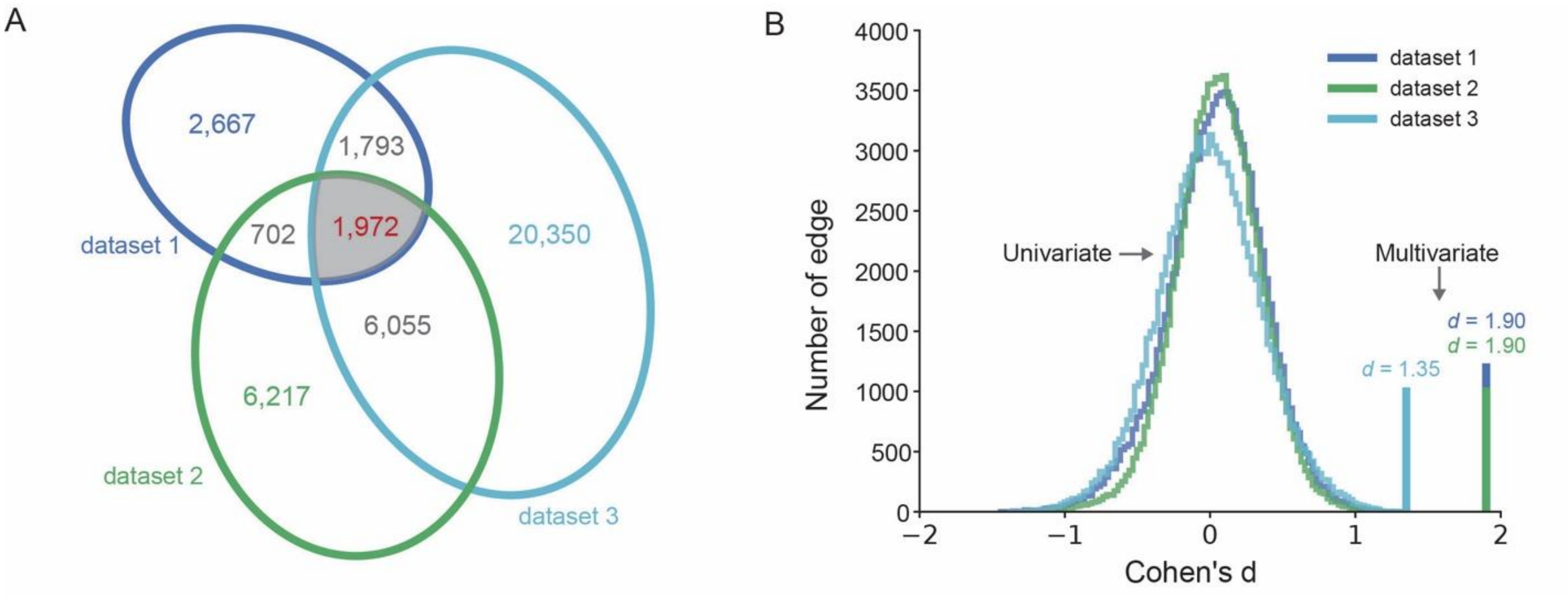

Supplementary Fig. 3. Comparison between univariate analysis and multivariate pattern analysis in detecting the effects of sleep deprivation. A. The number of significant functional connections (sleep-deprived vs. well-rested) identified across datasets after false discovery rate (FDR) correction. Of the connections identified as significant within each dataset, only 1,972 were consistently significant across datasets 1–3, representing a small proportion: 27.6% in dataset 1 (total: 7,134), 13.2% in dataset 2 (total: 14,946), and 6.5% in dataset 3 (total: 30,170). **B.** Effect sizes (Cohen's *d*) estimated using univariate and multivariate pattern analysis (MVPA) for sleep-deprived versus well-rested states. For univariate analysis, distributions of effect sizes for individual connections are shown. For MVPA, effect sizes were estimated using the probability of being sleep-deprived (PoSD) scores in datasets 2 and 3, and the area under the curve (AUC) for classification performance was converted to Cohen's d for dataset 1. These comparisons illustrate the increased robustness and sensitivity of MVPA compared to univariate analysis. Source data are provided as a Source Data file.

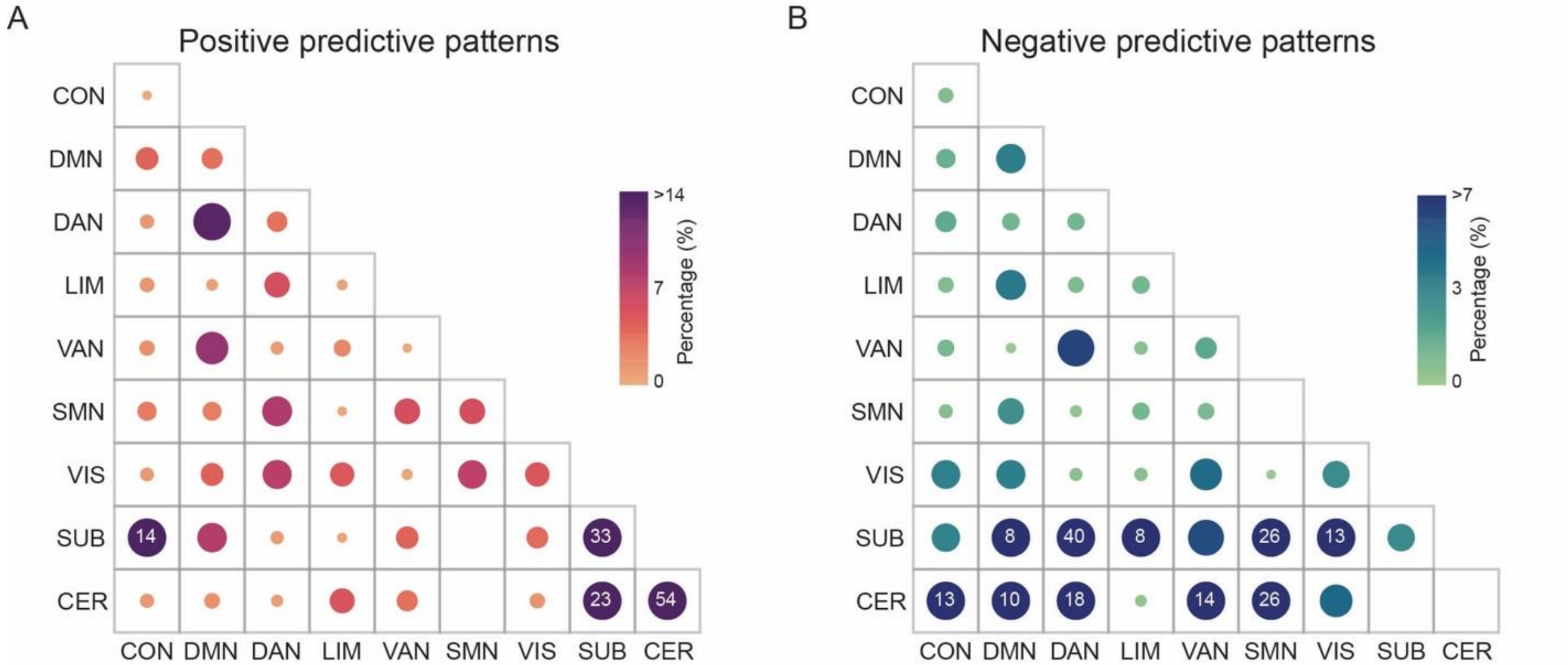

Supplementary Fig. 4. Percentages of significant connections within and between networks. A. Positive predictive patterns that were statistically significant. **B.** Negative predictive patterns that were statistically significant. Each cell indicates the percentage of connections within the corresponding network pair (indexed on the x- and y-axes) that were statistically significant based on bootstrap tests. The size and color intensity of each circle are proportional to the percentage value. For network pairs with exceptionally large values, e.g. between CER and SUB in panel **A**, the circle sizes and colors were capped, and the specific percentage values are labeled in the cells (e.g., 23% in this case). CON: frontoparietal control network; DMN: default mode network; VAN: ventral attention network; DAN: dorsal attention network; SMN: somatomotor network; VIS: visual network; LIM: limbic network; SUB: subcortical network; CEB: cerebellum network. Source data are provided as a Source Data file.
