# Supplementary material for: A neural signature of sleep deprivation in the human brain
Source: Nat Commun. 2026 Jul 22;17:8919. doi: 10.1038/s41467-026-75661-x (PMC13503868; doi:10.1038/s41467-026-75661-x)
Supplement: Supplementary file 2 [file 41467_2026_75661_MOESM2_ESM.pdf]

nature portfolio

Corresponding author(s): Mohammed R. Milad

Last updated by author(s): Jun 24, 2026

Reporting Summary

Nature Portfolio wishes to improve the reproducibility of the work that we publish. This form provides structure for consistency and transparency in reporting. For further information on Nature Portfolio policies, see our Editorial Policies and the Editorial Policy Checklist.

Statistics

For all statistical analyses, confirm that the following items are present in the figure legend, table legend, main text, or Methods section.

n/a	Confirmed	
☐	☒	The exact sample size (*n*) for each experimental group/condition, given as a discrete number and unit of measurement
☐	☒	A statement on whether measurements were taken from distinct samples or whether the same sample was measured repeatedly
☐	☒	The statistical test(s) used AND whether they are one- or two-sided<br>*Only common tests should be described solely by name; describe more complex techniques in the Methods section.*
☐	☒	A description of all covariates tested
☐	☒	A description of any assumptions or corrections, such as tests of normality and adjustment for multiple comparisons
☐	☒	A full description of the statistical parameters including central tendency (e.g. means) or other basic estimates (e.g. regression coefficient) AND variation (e.g. standard deviation) or associated estimates of uncertainty (e.g. confidence intervals)
☐	☒	For null hypothesis testing, the test statistic (e.g. *F*, *t*, *r*) with confidence intervals, effect sizes, degrees of freedom and *P* value noted<br>*Give P values as exact values whenever suitable.*
☒	☐	For Bayesian analysis, information on the choice of priors and Markov chain Monte Carlo settings
☒	☐	For hierarchical and complex designs, identification of the appropriate level for tests and full reporting of outcomes
☐	☒	Estimates of effect sizes (e.g. Cohen's *d*, Pearson's *r*), indicating how they were calculated

Our web collection on statistics for biologists contains articles on many of the points above.

Software and code

Policy information about availability of computer code

Data collection	No specific software was used during data collection.
Data analysis	Preprocessing of neuroimaging data was performed using fMRIPrep 20.0.2. Classification analysis was performed using scikit-learn 1.4.0 and nilearn 0.10.2. The codes to generate the main results are available at https://github.com/zhenfu-wen01/sleep_loss_signature.

For manuscripts utilizing custom algorithms or software that are central to the research but not yet described in published literature, software must be made available to editors and reviewers. We strongly encourage code deposition in a community repository (e.g. GitHub). See the Nature Portfolio guidelines for submitting code & software for further information.

Data

Policy information about availability of data

All manuscripts must include a data availability statement. This statement should provide the following information, where applicable:

- Accession codes, unique identifiers, or web links for publicly available datasets
- A description of any restrictions on data availability
- For clinical datasets or third party data, please ensure that the statement adheres to our policy

The functional connectivity data of Dataset 1 are available at https://osf.io/ayj72/. The raw data are not publicly available due to a lack of current institutional authorizations for public data sharing. The raw data could be available upon request and engage in a data-sharing agreement with the institution. Requests for Dataset 1 should be directed to A.G. (anne.germain.phd@gmail.com). Dataset 2 is available at https://osf.io/ubhpx/. Dataset 3 is from the Perturbation of the Depression Connectome (PDC) 1.0 data release at https://www.humanconnectome.org/. Dataset 4 is available in OpenNeuro database with accession number

ds000201. Dataset 5 is available at NIMH Data Archive through collection ID 2393. Dataset 6 is available from UK Biobank, which can be accessed by applying for access via its Access Management System at https://www.ukbiobank.ac.uk/enable-your-research/apply-for-access.

Research involving human participants, their data, or biological material

Policy information about studies with human participants or human data. See also policy information about sex, gender (identity/presentation), and sexual orientation and race, ethnicity and racism.

Reporting on sex and gender	Sex was self-reported by participants. Sex/gender were not a primary focus of this study, thus no sex- or gender-based analyses were conducted.
Reporting on race, ethnicity, or other socially relevant groupings	No socially constructed or socially relevant categorization variable was used in this study.
Population characteristics	Age and sex were counterbalanced in the discovery dataset across the three sleep manipulation conditions, thus they were not included as covariate variables. Head motion, age, and sex were included as covariate variables in the regression analysis and analysis of covariance (ANCOVA).
Recruitment	Participants were recruited via advertisements posted on online platforms and public transportation advertisements.
Ethics oversight	The study for the discovery dataset was approved by the University of Pittsburgh Institutional Review Board and the Department of Defense Human Research Protection Office. Studies if datasets 2-6 were approved by the Institutional Review Boards of the corresponding research sites as stated in previous publications. All participants gave informed consent before attending the corresponding study.

Note that full information on the approval of the study protocol must also be provided in the manuscript.

Field-specific reporting

Please select the one below that is the best fit for your research. If you are not sure, read the appropriate sections before making your selection.

[x] Life sciences [ ] Behavioural & social sciences [ ] Ecological, evolutionary & environmental sciences

For a reference copy of the document with all sections, see nature.com/documents/nr-reporting-summary-flat.pdf

Life sciences study design

All studies must disclose on these points even when the disclosure is negative.

Sample size	No sample-size calculation was performed. Sample sizes was based on the availability of existing study data. As far as we know, the sample size in this study is the largest in the field of sleep deprivation.
Data exclusions	Participants were included if their structural and functional images were available.
Replication	The replication and generalization of the findings were confirmed by successful classification of different sleep manipulation states on external validation datasets that were never used during model training.
Randomization	Participants of dataset 1 were randomized to 1 of 3 sleep conditions (normal sleep, sleep deprivation, or partial sleep deprivation) using a permuted block design, stratified by age and sex. Participants of dataset 4 were randomized to either first receive partial sleep deprivation or normal sleep. No randomization was performed for dataset 2, dataset 3, dataset 5, and dataset 6 regarding sleep conditions.
Blinding	Investigators were binded to group allocation during data collection.

Reporting for specific materials, systems and methods

We require information from authors about some types of materials, experimental systems and methods used in many studies. Here, indicate whether each material, system or method listed is relevant to your study. If you are not sure if a list item applies to your research, read the appropriate section before selecting a response.

Materials & experimental systems

n/a	Involved in the study
☒	☐ Antibodies
☒	☐ Eukaryotic cell lines
☒	☐ Palaeontology and archaeology
☒	☐ Animals and other organisms
☒	☐ Clinical data
☒	☐ Dual use research of concern
☒	☐ Plants

Methods

n/a	Involved in the study
☒	☐ ChIP-seq
☒	☐ Flow cytometry
☐	☒ MRI-based neuroimaging

Plants

Seed stocks: NA

Novel plant genotypes: NA

Authentication: NA

Magnetic resonance imaging

Experimental design

Design type: Both task and resting-state functional MRI were collected. Event-related design was used for task fMRI.

Design specifications: This study focused on intrinsic functional connectivity, thus the task activations during the experiments were not analyzed.

Behavioral performance measures: No behavioral performance analysis was performed.

Acquisition

Imaging type(s): Both structural and functional MRI data were collected.

Field strength: 3 Tesla.

Sequence & imaging parameters:

Dataset 1: High-resolution T1-weighted (T1w) structural images were acquired using a magnetization-prepared rapid gradient echo (MPRAGE) sequence (slice number = 192, repetition time [TR] = 2200 ms, echo time [TE] = 3.31 ms, flip angle = 9°, field of view [FOV] = 256 × 192 mm2, voxel size = 0.9 mm × 0.9 mm × 1.0mm3). Functional MRI images were collected using a gradient echo-planar imaging (EPI) sequence (slice number = 39, TR = 2000 ms, TE = 28 ms, flip angle = 90°, FOV = 20.5 mm2, matrix = 64 × 64, thickness = 3.1 mm).
Dataset 2: A multiband gradient-echo EPI sequence was used for resting-state data collection (TR = 2000 ms, TE = 24 ms, flip angle = 90°, matrix = 64 × 64 × 36, slice thickness = 4 mm). The resting-state run for each scanning session lasted 7 minutes. A high-resolution (0.8 × 0.8 × 0.8 mm) T1w anatomical scan was collected after the resting-state run.
Dataset 3: Description of imaging parameters was detailed at Harms et al. 2018. Briefly, The fMRI scans were acquired with a 2D multiband gradient-recalled echo echo-planar imaging sequence (MB8, TR/TE=800/37 ms, flip angle = 52°).
Dataset 4: Functional images were acquired using the following settings: TR = 2500 ms, TE = 30 ms, flip angle = 75°, slice thickness = 3 mm, slice number = 49, field of view = 28.8 cm. T1w images were acquired with a sagittal BRAVO sequence (field of view = 24 cm, slice thickness = 1 mm).
Dataset 5: High-resolution T1-weighted (T1w) structural images were acquired using a MPRAGE sequence. Functional MRI images were collected using a gradient echo-planar imaging sequence (slice number = 48, TR = 2560 ms, TE = 30 ms, flip angle = 90°, voxel size = 3 × 3 × 3 mm).
Dataset 6: Functional images were collected using the following parameters: multi-band = 8, TR = 735 ms, TE = 39 ms, flip angle = 52°, voxel size = 2.4 × 2.4 × 2.4 mm.

Area of acquisition: Whole brain.

Diffusion MRI: ☐ Used ☒ Not used

Preprocessing

Preprocessing software: Imaging data were preprocessed using fMRIPrep 20.0.2.

Normalization	Functional images were spatially normalized into the standard space through nonlinear registration.
Normalization template	The MNI152NLin2009cAsym template.
Noise and artifact removal	The following nuisance covariates, including six head motion parameters, averaged white matter signal, averaged cerebral spinal fluid (CSF) signal, their temporal derivatives, quadratic terms, and squares of derivatives were regressed out from the brain signals. The global signals were also regressed out following previous sleep deprivation studies.
Volume censoring	Brain volumes with framewise displacement (FD) larger than 0.3 mm were censored to reduce the impacts of high-motion volumes. Functional runs with mean FD larger than 0.5 were removed for further analysis.

Statistical modeling & inference

Model type and settings	Functional connectivity was modeled using the regional time series and Pearson's correlation. Predictive analyses were conducted to classify different sleep manipulation conditions, i.e., sleep deprivation vs. normal sleep.
Effect(s) tested	We estimated the classification accuracy (sleep deprivation vs. normal sleep) using cross-validation procedure on dataset 1. The generalizability of the constructed model were evaluated using force-choice classification accuracy on external datasets.

Specify type of analysis: ☒ Whole brain ☐ ROI-based ☐ Both

Statistic type for inference (See Eklund et al. 2016)	Permutation test was performed to test the significance of cross validation classification performance. Two-sided binomial tests were used to evaluate the significance of the forced-choice accuracies. Bootstrap tests were performed to identify the connections that significantly contributed to the classification model.
Correction	When applicable, FDR correction was performed.

Models & analysis

n/a	Involved in the study
☐	☒ Functional and/or effective connectivity
☒	☐ Graph analysis
☐	☒ Multivariate modeling or predictive analysis

Functional and/or effective connectivity	Pearson correlation was used to estimate functional connectivity.
Multivariate modeling and predictive analysis	Whole-brain functional connections were used as features, the support vector machine with linear kernel from scikit-learn was used as classifier. No feature selection/reduction was performed. Cross-validation procedure was performed on the discovery dataset to evaluate classification performance. A model was further trained with all data from the discovery dataset 1 for further generalization tests by applying the trained model to external validation datasets without any additional tuning of model parameters.
