# Supplementary material for: A neural signature of sleep deprivation in the human brain
Source: Nat Commun. 2026 Jul 22;17:8919. doi: 10.1038/s41467-026-75661-x (PMC13503868; doi:10.1038/s41467-026-75661-x)
Supplement: Supplementary file 3 [file 41467_2026_75661_MOESM3_ESM.pdf]

nature portfolio

Peer Review File

A neural signature of sleep deprivation in the human brain

Corresponding Author: Dr Mohammed Milad

This file contains all reviewer reports in order by version, followed by all author rebuttals in order by version.

Version 0:

Reviewer comments:

Reviewer #1

(Remarks to the Author)
A substantial body of neuroimaging studies has examined the neural consequences of sleep deprivation and short sleep duration. This paper stands out by externally validating a machine learning classifier across multiple datasets, despite differences in scanning protocols, preprocessing strategies, and task contexts. The findings are consistent with previous literature on sleep deprivation and sleep duration, and they provide additional confidence in the robustness of the results. What surprised me most is that the classifier was validated in datasets 2 and 3, which included patients with major depression and treatment-resistant depression—populations in which sleep deprivation is known to have acute antidepressant effects. I recommend that the authors further investigate whether there are group differences in classifier performance across these populations and using different colors to distinguish patients and HC in Figure 1C and 1D could help clarify any such differences.
Additionally, the sleep restriction condition in this study was limited to the second half of the night, which primarily impacts REM sleep. This design mirrors real-life situations where shortened sleep due to work schedules often affects the latter half of the night. Thus, the observed similarity in predictive patterns between full sleep deprivation and sleep restriction is both expected—given the overlap in affected sleep stages—and somewhat unexpected, considering that slow-wave sleep, which predominates in the first half of the night, plays a distinct role in restorative processes and might have different neural signature.
I also recommend that the authors, when feasible, further validate the association with total sleep time (TST) in the ABCD participants—who are younger than those in the discovery dataset—using the same approach employed for the UK Biobank sample.

Reviewer #2

(Remarks to the Author)
This article reports the fMRI analyses of multiple datasets that used a sleep restriction or sleep deprivation paradigm (with the exception of the UK biobank dataset), with the aim of identifying a consistent neural pattern of sleep deprivation using a multivariate pattern analysis (MVPA) approach. The authors identified a functional connectivity pattern associated with sleep deprivation from a discovery dataset, which they validated independent datasets. I had the opportunity to review a previous version of the manuscript that was submitted to another journal. The authors made some improvements, in particular providing more information on the details of each protocol and clarifying the methods of vigilance monitoring for some of the datasets. However, major concerns still persist, in relation to the large heterogeneity of the conditions under which these data were collected across the datasets, in particular:
- fMRI protocol: the discovery dataset was collected in task-based fMRI session of a threat conditioning paradigm, while other datasets were acquired in a resting-state; resting-state protocols themselves may vary a lot in instruction, stimuli or lack thereof, which were not clearly outlined;
- age of participants: dataset 4 included older adults, while the others included young to middle-aged individuals;
- their health status: datasets 2 and 3 included participants with major depression, and it is unclear whether some were medicated or not, and some of them may have received specific treatments such as ECT.
Such major differences question whether the reported signature is related to sleep deprivation/restriction or rather to some other aspects (e.g., emotional dysregulation) that could be amplified by a variety of factors including a lack of sleep.
It is important to note that the study was not preregistered, which constitutes a limitation to the transparency of this analysis.

Reviewer #3

(Remarks to the Author)
The authors build a functional connectivity based model that predicts sleep deprivation. Strengths of the paper include extensive external validation (testing the model on both within- and between-subjects effects) and clear writing. Although I am generally enthusiastic about the work because of the creative inclusion of multiple validation datasets, I have several major concerns. These include missing detail in the main text that makes it difficult to evaluate the robustness of the approach and predictions, potential influence of head motion, and lack of specificity tests. My specific comments are below.

Introduction.
The introduction is clear and well written, but insufficiently characterizes recent neuroimaging work on sleep in the brain. A number of recent papers have looked at large-scale network changes with sleep quantity and/or quality, predicted these measures within- and across-dataset with multivariate methods, and examined large datasets including the Adolescent Brain Cognitive Development Study and Human Connectome Project datasets. I recommend toning down the claims of novelty along these dimensions and focusing on the new approaches and insights that this work offers, such as generalization to sleep manipulation designs and isolation of sleep from other individual differences that may vary with a typical night's sleep by using random group assignment in the discovery dataset.

As a more minor point, it seems that the introduction uses “MVPA” to mean “machine learning”. Because MVPA is frequently used to refer to classification with voxelwise activity patterns, I was unsure at this point in the paper what the features used for prediction would be.

Results.
(1) Neural signature of sleep deprivation.
I appreciate the concise writing but the results lack sufficient detail. Because key information is only included in the methods, I was unsure about the robustness of the main model results until I got to that section at the end of the paper. For example, the results should mention how much fMRI data was collected per person, whether it was rest or task data, and what the general cross-validation procedure was (feature selection parameter tuning, train/test splits).

Similarly, head motion is one of the biggest concerns in work relating functional connectivity to sleep because both are related to motion, but the control analyses are only described in the supplement. I suggest providing some details about head motion controls in the main text given that they will be at the forefront of many readers' minds. For example, did head motion or amount of usable data after frame censoring differ between groups? Were model predictions in held-out people related to their sleep when controlling for head motion but not their head motion when controlling for their sleep?

Cross-validation in the discovery dataset will produce a different model for every cross-validation fold. How was a final “consensus model” defined for application to the two independent validation datasets? This should be mentioned in the results so it's always clear what model is being applied to what data.

For the external validation datasets (n=68, n=54, n=78), did mean frame-to-frame head motion or amount of usable data differ between pre- vs post-sleep deprivation states? Are prediction results robust to these potential confounds? For all external validations, do analyses rule out the possibility that the classifier is sensitive to time of day (morning vs. night) as opposed to sleep deprivation?

(2) Expression of the signature under other sleep manipulations
Can the authors confirm that the model tested on the post-recovery scans was trained only on data from separate participants (i.e., not the same participants whose recovery session was tested)? I think this is the case after reading the methods but this should be clarified in the results.

When PoSD scores are compared between groups in the discovery set, how are those scores generated? If they are generated from a final “consensus model”, this compromises independence between training and test data because participants used in model training (in the deprivation and normal sleep groups) are also included in the test set. If they are generated with different predictive models (i.e., defined on 90% of subjects and tested on the held-out 10%) this makes it difficult to compare PoSD scores between groups because those scores were generated with different models. Please clarify.

(3) Association with natural sleep variance
Please provide more details about the external validation datasets in the results. For example, were all participants adults? How were actigraphy data collected and processed? The lack of basic information about the data, participants, and measures makes it difficult to evaluate the strength of the validation evidence when reading the results.

The generalization to UK Biobank data is an excellent demonstration of model sensitivity but misses a critical opportunity to test model specificity. Are model predictions more closely related to measures of sleep time than other measures such as physical activity, stress, and cognitive performance? While these measures aren't independent of sleep, a model built to predict sleep should predict sleep measures more accurately than others. Specificity tests (at least in UK Biobank and ideally in other samples as well) are necessary to demonstrate that the neural signature is one of sleep and not related measures.

Another important type of specificity test is one about the specificity of predictive power to the model's features and weights.

For example, does the proposed neural signature predict sleep deprivation and individual differences in sleep better than, e.g., the strength of resting-state networks such as DMN and same-sized random networks?

I am interested in hearing more about what specific construct the authors think their model captures. Is it sleep deprivation per se or could it be something related to lack of sleep, such as low mood, low arousal or alertness, stress, frustration, effortful attempts to stay still and awake in the scanner, etc.? It could increase the theoretical impact of the results to test this by, for example, relating sleep model predictions to other measures and relating other neural signatures (e.g., of negative mood, arousal [https://journals.plos.org/ploscompbiol/article/authors?id=10.1371/journal.pcbi.1012994], or alertness [https://elifesciences.org/articles/62376]) to sleep.

(4) Neural patterns under other sleep manipulations
I did not fully follow the significance of the analyses in this section. What is the motivation for testing effects on features with positive vs. negative models weights separately?

Methods.
In the discovery dataset, data from different tasks (not rest) were concatenated to create a single functional connectivity matrix per person. Did task performance and/or the relative ratio of different tasks differ between groups? Are predictions robust to these potential confounds? If task performance did differ between sleep conditions and groups, this makes a specificity test showing that the sleep models do not predict cognitive performance better than they predict sleep even more important.

Signed,
Monica Rosenberg

Version 1:

Reviewer comments:

Reviewer #1

(Remarks to the Author)
Responses: Regarding Figure 1C and 1D, since the classifier performed similarly across groups, we retained a unified color scheme to preserve visual clarity. However, if the reviewer prefers, we are happy to revise the figures to distinguish patients and healthy controls with separate colors.

Comments: Thank you for the reply. Using a unified color scheme to preserve visual clarity is understandable. However, given a trend of significant group differences observed in dataset 3 ($p=0.09$), I recommend reporting separate descriptive values for PoSD changes in patients and controls in addition to t values.

Reviewer #2

(Remarks to the Author)
I appreciate the authors' clarifications, however Table S1 (which should be included in the main text because it conveys critical information to understand the study) clearly shows that the majority of validation datasets used resting state fMRI (all but one) and/or within-subject design. In these conditions, how can the authors justify the use of a task-based fMRI between-subject dataset as their training model ? This should be explicitly discussed.
Why not using a resting-state dataset for training as this may be more generalizable ?

Reviewer #3

(Remarks to the Author)
The revision thoroughly and thoughtfully addresses my comments. As one final suggestion, the authors may want to consider moving the arousal findings to the results. They are informative about what process(es) model predictions may reflect and it seems out of place to introduce new results in the discussion.
- Monica Rosenberg

Open Access This Peer Review File is licensed under a Creative Commons Attribution 4.0 International License, which permits use, sharing, adaptation, distribution and reproduction in any medium or format, as long as you give appropriate credit to the original author(s) and the source, provide a link to the Creative Commons license, and indicate if changes were made.
In cases where reviewers are anonymous, credit should be given to 'Anonymous Referee' and the source.
The images or other third party material in this Peer Review File are included in the article's Creative Commons license, unless indicated otherwise in a credit line to the material. If material is not included in the article's Creative Commons license and your intended use is not permitted by statutory regulation or exceeds the permitted use, you will need to obtain permission directly from the copyright holder.
To view a copy of this license, visit https://creativecommons.org/licenses/by/4.0/

Dear Editor and Reviewers,

We herein submit our revised manuscript "A neural signature of sleep deprivation in the human brain" to be considered for publication in Nature Communications. We are grateful to the reviewers and editor for your careful read of the paper and their insightful and constructive comments. We have revised the manuscript extensively in response to the comments, including adding new analyses, clarifying methodological details, and refining the interpretation of our findings. Below, we provide a point-by-point response to each comment and indicate the corresponding changes made in the manuscript (marked in red).

Response to Reviewer #1

Comment: *A substantial body of neuroimaging studies has examined the neural consequences of sleep deprivation and short sleep duration. This paper stands out by externally validating a machine learning classifier across multiple datasets, despite differences in scanning protocols, preprocessing strategies, and task contexts. The findings are consistent with previous literature on sleep deprivation and sleep duration, and they provide additional confidence in the robustness of the results.*

Response: We are immensely grateful for the positive feedback, and sincerely appreciate the insightful comments and suggestions. We have made a sincere effort to carefully address your requests, suggestions, and concerns point-to-point as detailed below.

Comment: *What surprised me most is that the classifier was validated in datasets 2 and 3, which included patients with major depression and treatment-resistant depression—populations in which sleep deprivation is known to have acute antidepressant effects. I recommend that the authors further investigate whether there are group differences in classifier performance across these populations and using different colors to distinguish patients and HC in Figure 1C and 1D could help clarify any such differences.*

Response: We thank the reviewer for this insightful suggestion. Datasets 2 and 3 were the only publicly available datasets on sleep deprivation that we found, and they included both healthy controls and patients with depression. Following the reviewer's recommendation, we conducted additional analyses comparing changes in PoSD (sleep deprivation minus normal sleep) between patients and controls. These analyses revealed no significant group differences in in either dataset (dataset 2: t = -1.38, p = 0.172; dataset 3: t = 1.69, p = 0.098), indicating that the neural signature captures sleep deprivation-related changes similarly across clinical and non-clinical populations. We added the following clarification to the Results section.

"Since datasets 2 and 3 contain both controls and patients with major depression or treatment resistant depression, we further compared PoSD changes from rested state to sleep-deprived state between controls and patients. We found no significant group differences in dataset 2 (t = -1.38, p = 0.172) and dataset 3 (t = 1.69, p = 0.098),

suggesting that the neural signature of sleep deprivation generalizes across clinical and non-clinical populations.**”**

Regarding Figure 1C and 1D, since the classifier performed similarly across groups, we retained a unified color scheme to preserve visual clarity. However, if the reviewer prefers, we are happy to revise the figures to distinguish patients and healthy controls with separate colors.

Comment: *Additionally, the sleep restriction condition in this study was limited to the second half of the night, which primarily impacts REM sleep. This design mirrors real-life situations where shortened sleep due to work schedules often affects the latter half of the night. Thus, the observed similarity in predictive patterns between full sleep deprivation and sleep restriction is both expected—given the overlap in affected sleep stages—and somewhat unexpected, considering that slow-wave sleep, which predominates in the first half of the night, plays a distinct role in restorative processes and might have different neural signature.*

Response: We thank the reviewer for this thoughtful comment. We agree that the timing of sleep restriction in our study, which disproportionately impacts REM sleep, likely contributed to the observed similarity between full sleep deprivation and partial sleep restriction. We have now emphasized this point in the Discussion and noted that future studies directly comparing first-half versus second-half restriction could help disentangle the differential contributions of slow-wave sleep and REM sleep to brain network disruptions. The following contents were added to the Discussion section.

“It is important to note that the sleep restriction condition in this study was limited to the second half of the night, disproportionately impacting REM sleep. This design choice mirrors real-world situations in which shortened sleep often occurs during the latter part of the night. The similarity in predictive patterns between full sleep deprivation and partial sleep restriction may therefore reflect the shared reduction in REM-rich sleep. At the same time, slow-wave sleep, which predominates in the first half of the night and plays a key role in restorative processes, may have yielded a different neural signature if selectively restricted. Future studies that directly compare first-half versus second-half sleep restriction could further elucidate the distinct contributions of REM and slow-wave sleep to the neural consequences of sleep loss.”

Comment: *I also recommend that the authors, when feasible, further validate the association with total sleep time (TST) in the ABCD participants—who are younger than those in the discovery dataset—using the same approach employed for the UK Biobank sample.*

Response: We appreciate the reviewer's thoughtful suggestion to extend the analysis to the ABCD cohort. Unfortunately, we do not currently have access to the ABCD data required to perform these analyses. This has become more challenging because the first author recently transferred institutions (out of the United States), which necessitates re-establishing data access agreements. We totally agree that testing developmental generalizability is important, and we now note this explicitly in the Discussion as a key future direction.

"An important future direction is to examine whether the sleep-deprivation neural signature generalizes to younger populations. Large developmental datasets such as the adolescent brain cognitive development (ABCD) study[76] could help determine whether PoSD relates to habitual sleep variation earlier in life. Future work leveraging developmental cohorts will be essential for understanding how the neural expression of sleep loss varies across the lifespan."

Reference: 76. Casey, B. J. *et al.* The adolescent brain cognitive development (ABCD) study: imaging acquisition across 21 sites. *Dev. Cogn. Neurosci.* **32**, 43–54 (2018).

Response to Reviewer #2

Comment: *This article reports the fMRI analyses of multiple datasets that used a sleep restriction or sleep deprivation paradigm (with the exception of the UK biobank dataset), with the aim of identifying a consistent neural pattern of sleep deprivation using a multivariate pattern analysis (MVPA) approach. The authors identified a functional connectivity pattern associated with sleep deprivation from a discovery dataset, which they validated independent datasets. I had the opportunity to review a previous version of the manuscript that was submitted to another journal.*

The authors made some improvements, in particular providing more information on the details of each protocol and clarifying the methods of vigilance monitoring for some of the datasets. However, major concerns still persist, in relation to the large heterogeneity of the conditions under which these data were collected across the datasets, in particular:

- fMRI protocol: the discovery dataset was collected in task-based fMRI session of a threat conditioning paradigm, while other datasets were acquired in a resting-state; resting-state protocols themselves may vary a lot in instruction, stimuli or lack thereof, which were not clearly outlined;

- age of participants: dataset 4 included older adults, while the others included young to middle-aged individuals;

- their health status: datasets 2 and 3 included participants with major depression, and it is unclear whether some were medicated or not, and some of them may have received specific treatments such as ECT.

Such major differences question whether the reported signature is related to sleep deprivation/restriction or rather to some other aspects (e.g., emotional dysregulation) that could be amplified by a variety of factors including a lack of sleep.

Response: We thank the reviewer for raising this important point. We agree that the datasets differ in scanning protocols, demographic composition, and health status. Below we clarify why these differences are unlikely to confound our main findings and, instead, provide a strong test of the generalizability of the neural signature.

1. The decoder was trained exclusively on dataset 1

The neural signature was developed only using dataset 1, which consists of healthy adults randomly assigned to different sleep conditions. None of the external datasets contributed to model training. Therefore, variations in experimental protocol or participant characteristics in datasets 2–6 cannot influence the learned model.

2. External validation datasets 2-4 contain within-subject comparisons

In datasets 2-4, each participant was scanned twice, once after a normal sleep and once after sleep deprivation or restriction. This within-individual cross-over design enables the decoder to be evaluated on state changes in PoSD scores within the same individual, thereby controlling for stable factors such as age, sex, clinical status, scanner, or protocol. The decoder successfully identified the sleep-deprived session in 83-94% of individuals across these datasets, indicating that its performance reflects neural changes due to sleep loss rather than other factors such as demographic or diagnostic variation.

3. Covariates were controlled when linking PoSD and total sleep time in datasets 5-6

For the observational datasets with one fMRI session (datasets 5–6), all analyses controlled for age, sex, site, and head motion. Associations between PoSD and total sleep time remained significant after covariate adjustment, indicating that demographic variables and motion do not account for the observed effects.

Overall, these points suggest that our results are unlikely to be driven by factors other than sleep deprivation. Moreover, the ability of the neural signature to generalize across diverse acquisition protocols, demographic groups, and clinical populations supports its robustness as a marker of sleep-related neural variation. Following the reviewer's comment, we have added a table summarizing key characteristics of each dataset to further clarify how these differences relate to the analyses performed (Supplementary Table S1). We have also added the following text to the revised manuscript to clarify these points.

"A notable feature of the present study is the heterogeneity across datasets in acquisition protocols, demographic composition, and clinical characteristics. Rather than representing a limitation, this heterogeneity provides a strong test of the generalizability of the neural signature. Importantly, the model was trained on a randomized experimental dataset comprising healthy adults and subsequently evaluated in independent datasets using within-subject cross-over designs (datasets 2-4), enabling the isolation of state-dependent changes associated with sleep

manipulation while controlling for stable factors such as age, clinical status, and scanner-related variability. In cross-sectional datasets (datasets 5-6), associations between PoSD and total sleep time were assessed while controlling for potential confounds, including age, sex, head motion, and other cognitive and psychological factors. The consistent performance of the neural signature across these diverse datasets suggests that it captures sleep-related neural state changes that generalize across contexts."

Supplementary Table S1. Key characteristics of datasets used in the study.

Dataset	Role in study	Design type	Sample characteristics	Scanning protocol	Sleep manipulation	Analysis objective
Dataset 1	Model development	Randomized experimental (between-subject)	Sample: 151 healthy adults (69 male, age: 23.8 ± 3.3);	About 26 minutes task-based fMRI.	Randomized: normal sleep (n = 48), sleep deprivation (n = 52), sleep restriction (n = 51).	Train classifier to distinguish sleep deprivation vs. normal sleep
Dataset 2	External validation	Within-subject cross-over	Sample: 68 adults, including 30 major depression disorder (7 male, age: 34 ± 9.9) and 38 healthy control (21 male, 33.5 ± 8.6)	About 7 minutes resting-state fMRI from each session.	Two sessions: normal sleep vs. sleep deprivation	Classify sleep condition within individuals
Dataset 3	External validation	Within-subject cross-over	Sample: 54 adults (23 male, age: 30.6 ± 9.4), including 44 patients with treatment resistant depression and 10 healthy control.	About 23 minutes, combined task-based and resting-state fMRI from each session.	Two sessions: normal sleep vs. sleep deprivation	Classify sleep condition within individuals
Dataset 4	External validation	Within-subject cross-over	Sample: 78 healthy adults, including 42 younger (21 male, age: 20-30 years) and 36 older	About 16 minutes resting-state fMRI from each session.	Two sessions: normal sleep vs. sleep restriction	Classify sleep condition within individuals

			volunteers (17 male, age: 65-75 years).			
Dataset 5	External validation	Cross-sectional	Sample: 109 adults (40 male; age: 24.7 ± 5.0 years)	About 26 minutes task-based fMRI from one session.	No manipulation; Total sleep time from actigraphy (~2 weeks).	Correlate probability of being sleep-deprived (PoSD) with habitual sleep duration.
Dataset 6	External validation	Cross-sectional	Sample: 39,799 adults (age: 40-69)	About 6 min. resting-state fMRI from one session.	No manipulation; Total sleep time estimated from self-report and accelerometry.	Correlate PoSD with habitual sleep duration.

Comment: *It is important to note that the study was not preregistered, which constitutes a limitation to the transparency of this analysis.*

Response: We agree with the reviewer that preregistration enhances transparency and reduces analytical flexibility. This study was not preregistered, and we now acknowledge this explicitly as a limitation in the Discussion. Importantly, the analytic pipeline was fixed prior to validation and applied unchanged to multiple independent datasets, including within-subject sleep manipulation samples, which mitigates concerns about analytic flexibility. A statement has been added to clarify this.

"Fourth, this study was not preregistered, which limits full analytical transparency. However, the primary analyses used a fixed training pipeline applied consistently across multiple independent datasets, including within-subject sleep manipulation samples, reducing analytical flexibility. Future work would benefit from formal preregistration."

Response to Reviewer #3

Comment: *The authors build a functional connectivity based model that predicts sleep deprivation. Strengths of the paper include extensive external validation (testing the model on both within- and between-subjects effects) and clear writing. Although I am generally enthusiastic about the work because of the creative inclusion of multiple validation datasets, I have several major concerns. These include missing detail in the main text that makes it difficult to evaluate the robustness of the approach and predictions, potential influence of head motion, and lack of specificity tests. My specific comments are below.*

Response: We are immensely grateful for the positive feedback, and sincerely appreciate the insightful comments and suggestions. We have made a sincere effort to carefully address your requests, suggestions, and concerns point-to-point as detailed below.

Introduction.

Comment: *The introduction is clear and well written, but insufficiently characterizes recent neuroimaging work on sleep in the brain. A number of recent papers have looked at large-scale network changes with sleep quantity and/or quality, predicted these measures within- and across-dataset with multivariate methods, and examined large datasets including the Adolescent Brain Cognitive Development Study and Human Connectome Project datasets. I recommend toning down the claims of novelty along these dimensions and focusing on the new approaches and insights that this work offers, such as generalization to sleep manipulation designs and isolation of sleep from other individual differences that may vary with a typical night's sleep by using random group assignment in the discovery dataset.*

Response: We thank the reviewer for this insightful comment. We agree that several recent studies have applied multivariate approaches to characterize brain-sleep relationships. To better situate our work within this growing literature, we have revised the Introduction to acknowledge these contributions and to clarify that our methodological contribution lies not in the application of multivariate modeling per se, but in extending these predictive frameworks to experimentally manipulated sleep paradigms and their generalization across sleep manipulation designs. The revised contents are as below.

“A powerful methodology for inference at the individual level is machine learning-based multivariate prediction[29–32], which integrates neural patterns across distributed circuits such as whole-brain connectivity measures to construct predictive models that distinguishing between different mental state. The resulting models, often referred to as decoders or neural signatures, offer higher sensitivity and reproducibility compared to traditional univariate analyses[21,33,34]. Using multivariate prediction techniques, recent studies have predicted individual differences in sleep quantity and/or quality from large-scale neuroimaging datasets[35,36]. These works have significantly advanced understanding of how large-scale neural organization reflects individual variation in sleep behavior. However, most of these studies have been linking brain measures to spontaneous variability in sleep rather than experimentally manipulating sleep. This makes it difficult to isolate the direct neural consequences of acute sleep loss from other factors that may co-vary with habitual sleep duration or quality.

Building on this progress, we aimed to extend multivariate prediction approaches to controlled sleep manipulation paradigms, which allow direct characterization of the neural consequences of acute sleep loss while minimizing individual confounds through random group assignment to sleep conditions. A key advantage of predictive modeling is that the constructed decoders can generalize across contexts[24,32], enabling the evaluation of neural signature expression under varying conditions, such as sleep deprivation, recovery sleep, or habitual sleep. In the case of sleep deprivation, such a signature may capture both transient disruptions induced by sleep loss in neural systems and the residual perturbations that persist after recovery sleep, providing insights into the dynamic effects of sleep manipulation. However, to-date, a sensitive and generalizable neural signature for sleep deprivation has yet to be established.

Here, we extend recent studies linking brain connectivity and sleep to experimentally manipulated sleep paradigms, isolating the neural consequences of acute sleep loss.”

Reference:
21. Marek, S. *et al.* Reproducible brain-wide association studies require thousands of individuals. *Nature* 603, 654–660 (2022).
24. Woo, C.-W., Chang, L. J., Lindquist, M. A. & Wager, T. D. Building better biomarkers: brain models in translational neuroimaging. *Nat. Neurosci.* 20, 365–377 (2017).
29. Chang, L. J., Gianaros, P. J., Manuck, S. B., Krishnan, A. & Wager, T. D. A sensitive and specific neural signature for picture-induced negative affect. *PLOS Biol.* 13, e1002180 (2015).
30. Rosenberg, M. D. *et al.* A neuromarker of sustained attention from whole-brain functional connectivity. *Nat. Neurosci.* 19, 165–171 (2016).

31.Wager, T. D. *et al.* An fMRI-based neurologic signature of physical pain. *N. Engl. J. Med.* 368, 1388–1397 (2013).
32. Shen, X. *et al.* Using connectome-based predictive modeling to predict individual behavior from brain connectivity. *Nat. Protoc.* 12, 506–518 (2017).
33. Kragel, P. A., Han, X., Kraynak, T. E., Gianaros, P. J. & Wager, T. D. Functional MRI can be highly reliable, but it depends on what you measure: a commentary on Elliott et al. (2020). *Psychol. Sci.* 32, 622–626 (2021).
34. Rosenberg, M. D. & Finn, E. S. How to establish robust brain–behavior relationships without thousands of individuals. *Nat. Neurosci.* 25, 835–837 (2022).
35. Mummaneni, A. *et al.* Functional brain connectivity predicts sleep duration in youth and adults. *Hum. Brain Mapp.* 44, 6293–6307 (2023).
36. Perrault, A. A. *et al.* Identification of five sleep-biopsychosocial profiles with specific neural signatures linking sleep variability with health, cognition, and lifestyle factors. *PLOS Biol.* 23, e3003399 (2025).

Comment: *As a more minor point, it seems that the introduction uses “MVPA” to mean “machine learning”. Because MVPA is frequently used to refer to classification with voxelwise activity patterns, I was unsure at this point in the paper what the features used for prediction would be.*

Response: We thank the reviewer for pointing out this ambiguity. To avoid confusion, we have replaced ‘MVPA’ with the more general term ‘predictive models’, and mentioned that such models are usually based on ‘whole-brain connectivity measures’. The revised text in the Introduction now reads:

“A powerful methodology for inference at the individual level is machine learning-based multivariate prediction[29–32], which integrates neural patterns across distributed circuits such as whole-brain connectivity measures to construct predictive models that distinguishing between different mental state.”

Reference:

29. Chang, L. J., Gianaros, P. J., Manuck, S. B., Krishnan, A. & Wager, T. D. A sensitive and specific neural signature for picture-induced negative affect. *PLOS Biol.* 13, e1002180 (2015).
30. Rosenberg, M. D. *et al.* A neuromarker of sustained attention from whole-brain functional connectivity. *Nat. Neurosci.* 19, 165–171 (2016).
31.Wager, T. D. *et al.* An fMRI-based neurologic signature of physical pain. *N. Engl. J. Med.* 368, 1388–1397 (2013).

32. Shen, X. *et al.* Using connectome-based predictive modeling to predict individual behavior from brain connectivity. *Nat. Protoc.* 12, 506–518 (2017).

Results.

Comment: *(1) Neural signature of sleep deprivation. I appreciate the concise writing but the results lack sufficient detail. Because key information is only included in the methods, I was unsure about the robustness of the main model results until I got to that section at the end of the paper. For example, the results should mention how much fMRI data was collected per person, whether it was rest or task data, and what the general cross-validation procedure was (feature selection parameter tuning, train/test splits).*

Response: We agree that key methodological details should be more clearly reported alongside the main findings. In the revised manuscript, we have added concise methodological descriptions (e.g., fMRI data amount per participant, rest vs. task context, and cross-validation procedure) directly within the Results section, while retaining the full details in the Methods. Please see below for some examples of the revised contents.

"All participants underwent three task-based fMRI sessions, a morning session (26 minutes of fMRI data) and an evening session (32 minutes of fMRI data) following sleep manipulation, and a morning session (32 minutes of fMRI data) the next day after recovery sleep (Fig. 1A, Methods)."

"These 97,461 connections were used as features (without feature selection) for a linear kernel support vector machine (SVM; default hyperparameter: C = 1.0)."

"First, we evaluated the classification performance using a 10 × 10 cross-validation procedure. Specifically, participants were randomly partitioned into 10 folds; in each iteration, one fold was held out as the test set while the remaining nine folds were used for model training. This cross-validation procedure was repeated 10 times with different random splits, and the mean performance across all 10 repetitions was reported."

Comment: *Similarly, head motion is one of the biggest concerns in work relating functional connectivity to sleep because both are related to motion, but the control analyses are only described in the supplement. I suggest providing some details about head motion controls in the*

main text given that they will be at the forefront of many readers' minds. For example, did head motion or amount of usable data after frame censoring differ between groups? Were model predictions in held-out people related to their sleep when controlling for head motion but not their head motion when controlling for their sleep?

Response: We thank the reviewer for highlighting the importance of head motion as a potential confound in functional connectivity analyses of sleep. We agree that providing more details about head motion control in the main text would be helpful for readers. In the revised manuscript, we have moved the description of the control analyses to the main text and expanded them. Specifically, we showed that in a motion-matched subset of participants from the discovery dataset, the classification performance remained significant (AUC = 0.90). We did not perform group comparisons on PoSD within the discovery dataset, as PoSD values derived from cross-validation are not fully independent across participants. Instead, we assessed prediction robustness to head motion using external validation datasets. In these datasets, the effect of sleep condition on PoSD remained significant after controlling for mean framewise displacement (FD) and the number of usable volumes as covariates (see response to related comment below). Together, these results indicate that model predictions are associated with sleep condition rather than head motion. We have incorporated these details into the main text to improve clarity.

"A commonly observed phenomenon in sleep deprivation studies is that head motion during scanning was higher in the sleep deprivation state compared to the normal sleep state. In the discovery dataset, we also observed significant differences between the sleep deprivation group and normal sleep group in mean framewise displacement (FD) ($t = 4.05$, $p = 1.04E-4$) and the number of usable volumes after censoring ($t = 3.51$, $p=6.88e-4$). To ensure that classification performance was not confounded by head motion, we performed additional analyses using a motion-matched subset of participants. Specifically, we excluded 10 participants with the highest FD measures from the sleep deprivation group and 10 participants with the lowest FD measures from the normal group, resulting in a subset with comparable motion metrics between groups (total n = 80; sleep deprivation: n = 42; normal sleep: n = 38; FD: $t = 1.03$, $p = 0.30$; usable volumes: $t = -1.06$, $p = 0.29$). We then conducted a 10 × 10 cross-validation procedure on this subset of participants. The resulting classification performance was comparable to the main analysis (AUC = 0.90, permutation test $p < 0.001$)."

Comment: *Cross-validation in the discovery dataset will produce a different model for every cross-validation fold. How was a final "consensus model" defined for application to the two independent validation datasets? This should be mentioned in the results so it's always clear what model is being applied to what data.*

Response: After cross-validation confirmed robust classification in the discovery dataset, we retrained a single final model using all participants from the discovery dataset (sleep deprivation and normal sleep groups). This final model was then applied to the independent validation datasets without further modification. We have clarified this procedure in the revised manuscript, which now reads:

"Next, we tested the generalizability of this neural signature by applying it to independent participants whose data had never been used in model training. A final decoder was retrained using all participants from the discovery dataset (sleep deprivation and normal sleep groups) and applied directly to the independent validation datasets without further modification."

Comment: *For the external validation datasets (n=68, n=54, n=78), did mean frame-to-frame head motion or amount of usable data differ between pre- vs post-sleep deprivation states? Are prediction results robust to these potential confounds? For all external validations, do analyses rule out the possibility that the classifier is sensitive to time of day (morning vs. night) as opposed to sleep deprivation?*

Response: We thank the reviewer for raising these important concerns. For the time-of-day effects, according to the original study protocols, scanning times were matched between the normal sleep and sleep deprivation sessions in all three external validation datasets. Therefore, the classifier is unlikely to distinguish time-of-day differences between sessions. We have clarified this point in the revised manuscript as follows:

"In addition, scanning times were matched between the normal sleep and sleep deprivation sessions in these datasets, minimizing potential confounding effects of time of day."

For concerns regarding head motion, we further examined potential confounds related to head motion in the external validation datasets. Motion metrics were available for datasets 3 and 4 (dataset 2 only provided preprocessed data without motion estimates). While small differences in mean framewise displacement (FD) and/or the number of usable volumes were observed between conditions in some datasets, the key findings remained robust when these variables were included as covariates. We added the following content to the revised manuscript.

"We further examined potential confounds related to head motion in dataset 3 and dataset 4, for which motion estimates were available. In dataset 3, mean framewise displacement (FD) was comparable between the normal sleep and sleep deprivation sessions ($t(53) = -1.66$, $p = 0.102$), while the number of usable volumes was slightly higher in the sleep deprivation session ($t(53) = 2.62$, $p = 0.011$). To ensure that the observed PoSD differences were not driven by head motion, we used a linear mixed-effects model with sleep condition as a fixed effect and participant as a random effect, while including mean FD and usable volumes as covariates. The effect of sleep condition on PoSD remained significant ($b = 0.331$, $z = 9.051$, $p = 1.41e-19$, 95%CI = [0.259, 0.403]). Similar results were observed in dataset 4, where mean FD ($t(77) = -1.69$, $p = 0.094$) and the number of usable volumes ($t(77) = -0.031$, $p = 0.975$) were comparable between normal sleep and sleep deprivation sessions. After controlling for mean FD and usable volumes, the effect of sleep condition on PoSD remained significant ($b = 0.110$, $z = 3.500$, $p = 4.66e-4$, 95%CI = [0.048, 0.172]). These results indicate that model predictions primarily reflect sleep condition rather than head motion."

Comment: *(2) Expression of the signature under other sleep manipulations*

Can the authors confirm that the model tested on the post-recovery scans was trained only on data from separate participants (i.e., not the same participants whose recovery session was tested)? I think this is the case after reading the methods but this should be clarified in the results.

Response: The reviewer is correct that the model tested on post-recovery scans was trained only on data from separate participants; that is, the post–sleep deprivation data of individuals whose recovery scans were tested were never included in model training. We have now made this explicit in the Results section to clarify it.

“Note that the decoders tested on post-recovery scans were trained on data from independent participants, so that data of individuals whose recovery scans were tested were never included in model training.”

Comment: *When PoSD scores are compared between groups in the discovery set, how are those scores generated? If they are generated from a final “consensus model”, this compromises independence between training and test data because participants used in model training (in the deprivation and normal sleep groups) are also included in the test set. If they are generated with different predictive models (i.e., defined on 90% of subjects and tested on the held-out 10%) this makes it difficult to compare PoSD scores between groups because those scores were generated with different models. Please clarify.*

Response: We thank the reviewer for raising this point and apologize for any confusion. In our original version, we did not directly compare PoSD scores between groups in the discovery dataset. We believe the reviewer’s comment is referring to the following sentence: “Using data from individuals in the sleep restriction group of dataset 1 (n = 51), we found that PoSD scores after sleep restriction were significantly higher than those after a night of recovery sleep (t = 5.41, p = 1.75E-6, Cohen’s d = 0.81, **Fig. 2B**).” Here, the PoSD score comparison was conducted within the sleep restriction group, i.e., comparing the same participants’ PoSD scores after sleep restriction versus after recovery sleep.

To clarify, the PoSD scores used in this analysis were computed using a final decoder trained exclusively on the sleep deprivation and normal sleep group. The sleep restriction group was not used in model training and therefore served as an independent test set. We have revised the text in the Results to explicitly note this independence to avoid confusion.

“Using data from individuals in the sleep restriction group of dataset 1 (n = 51), we tested whether PoSD differed between sleep restriction and recovery sleep. PoSD scores were derived using the final decoder trained on the sleep deprivation and normal sleep groups only, ensuring full independence between training and test data. We found that PoSD scores after sleep restriction were significantly higher than those after a night of recovery sleep (t = 5.41, p = 1.75E-6, Cohen’s d = 0.81, Fig. 2B).**”**

Regarding the sentence reporting the proportion of individuals with PoSD > 0.5 across groups (i.e., "Specifically, 35.3% of individuals had PoSD scores higher than 0.5, compared to 14.6% in the normal sleep group 14.6% and 82.7% in the sleep deprivation group."), this was intended to provide descriptive context rather than a formal statistical comparison. The proportions for the normal sleep and sleep deprivation groups were derived from cross-validation and therefore are not strictly independent of model training. We have clarified this point in the revised text to avoid misinterpretation.

"Specifically, 35.3% of individuals in the sleep restriction group had PoSD scores greater than 0.5. For descriptive comparison, the corresponding proportions based on cross-validation were 14.6% in the normal sleep group and 82.7% in the sleep deprivation group."

(3) Association with natural sleep variance

Comment: *Please provide more details about the external validation datasets in the results. For example, were all participants adults? How were actigraphy data collected and processed? The lack of basic information about the data, participants, and measures makes it difficult to evaluate the strength of the validation evidence when reading the results.*

Response: We thank the reviewer for this helpful suggestion. We have now added brief descriptions of key demographic and data collection information for each dataset in the Results section (e.g., whether participants were adults, relevant clinical status, and actigraphy collection/processing procedures), while retaining full details in the Methods. Some modifications in the main text are as below.

"To test this, we analyzed data from 109 young adults (dataset 5; age: 24.7 ± 5.0 years; 40 male, 69 female), who participated in an independent neuroimaging study. Each participant wore a wrist actigraphy device (Actiwatch-2) continuously for two weeks prior to their task-based fMRI session (26 minutes). Actigraphy data were processed using standardized pipelines to derive nightly TST, which was then averaged across all nights with usable data for each participant."

"In a subset of participants with accelerometer-based TST estimates available (n = 17,209; age: 64.0 ± 7.7 years; 7,700 male, 9,509 female), we further examined the association between PoSD and objectively measured sleep duration. Wrist-worn triaxial accelerometer data were

collected continuously for 7 days using the Axivity AX3 device and processed with a machine learning-based pipeline to derive TST, defined as the average proportion of time spent sleeping across the monitoring period.*”*

Comment: *The generalization to UK Biobank data is an excellent demonstration of model sensitivity but misses a critical opportunity to test model specificity. Are model predictions more closely related to measures of sleep time than other measures such as physical activity, stress, and cognitive performance? While these measures aren't independent of sleep, a model built to predict sleep should predict sleep measures more accurately than others. Specificity tests (at least in UK Biobank and ideally in other samples as well) are necessary to demonstrate that the neural signature is one of sleep and not related measures.*

Response: We thank the reviewer for this insightful comment. We agree that testing the specificity of the neural signature is critical to demonstrate that it reflects sleep-related variation rather than general behavioral or physiological factors. To address this, we conducted additional analyses in the UK Biobank dataset to examine whether PoSD was more strongly associated with sleep duration compared to other measures, including average daily physical activity, cognitive performance, substance use, and adverse childhood experiences. As shown in the Supplementary Information, correlations between PoSD and self-reported/accelerometer-based total sleep time (TST; $|r| = 0.067$ and 0.096) were consistently stronger than correlations with other measures ($|r| < 0.04$, Supplementary Table S2). And associations between PoSD and TST remained significant after controlling for the other measures as covariates. These findings suggest that the neural signature preferentially captures variance related to sleep, rather than reflecting general behavioral or physiological factors. We have added these analyses to the revised manuscript as below.

“To evaluate the specificity of PoSD, we examined its associations with additional behavioral and psychological measures in the UK Biobank dataset, including physical activity, cognitive performance, substance use, and adverse childhood experiences (Supplementary Results). Correlations between PoSD and these measures were relatively small ($|r| < 0.04$, Supplementary Table S2), and consistently lower than those observed for total sleep time ($|r| = 0.11–0.14$). The correlations between PoSD and TST remained significant after controlling for all the other measures ($|r| = 0.067-0.096$, $p < 1.60e-36$). Together, these results suggest that the neural

signature is more strongly associated with sleep-related variation than with other behavioral or cognitive measures."

Supplementary Table S2. Association between the probability of being sleep-deprived and different measures.

**Self-reported total sleep time**	**Accelerometer-based total sleep time**	**Matrix**	**Reaction time**	**Numeric memory**	**Symbol digit**	**Tower**
r = -0.067, p = 5.28E-41	r = -0.096, p = 3.96E-36	r = 0.020, p = 9.27e-4	r = -0.012, p = 0.021	r = 0.012, p = 0.054	r = 0.026, p = 1.60E-5	r = 0.014, p = 0.021
**Prospective memory**	**Fluid intelligence**	**Adverse childhood experience**	**Physical activity**	**Nicotine use**	**Cannabis use**	**Alcohol intake**
r = 0.005, p = 0.356	r = -0.013, p = 0.015	r = 0.007, p = 0.225	r = 0.008, p = 0.149	r = -0.005, p = 0.645	r = 0.006, p = 0.314	r = -0.040, p = 2.80E-11

The following content was added to the Supplementary Information.

"To evaluate the specificity of the neural signature, we examined the associations between the probability of being sleep-deprived (PoSD) and a range of behavioral and psychological measures in the UK Biobank cohort. Measures were defined following our prior work using the UK Biobank dataset. Cognitive measures were derived using tasks from the UK Biobank cognitive test battery, including reaction time, numeric memory, symbol digit substitution, matrix reasoning, tower rearranging, and prospective memory, which capture domains such as processing speed, working memory, reasoning, executive function, and fluid intelligence. Additional measures included fluid intelligence, physical activity (derived from the International Physical Activity Questionnaire – Short form), nicotine use (average number of pack-years smoked by an individual each year over adulthood), alcohol intake (by multiplying frequency of drinking alcohol and amount of alcohol drunk on a typical drinking day), cannabis use (total times of cannabis use in their lifetime), and adverse childhood experiences (based on questionnaire items adapted from the Childhood Trauma Screener).

For each participant, PoSD values were obtained by applying the trained decoder to resting-state functional connectivity data. Associations between PoSD and each measure were quantified using partial correlations, controlling for age, sex, scanning site, and head motion. Results are summarized alongside correlations between PoSD and self-reported and accelerometer-based total sleep time in Supplementary Table S2. To further assess specificity, we conducted additional partial correlation analyses to test whether associations between PoSD and total sleep time remained significant when all other measures were included as covariates. These analyses yielded similar results as in our main analysis (self-reported: $r = -0.067$, $p = 1.05E-40$; accelerometer-based: $r = -0.096$, $p = 1.59E-36$)."

Comment: *Another important type of specificity test is one about the specificity of predictive power to the model's features and weights. For example, does the proposed neural signature predict sleep deprivation and individual differences in sleep better than, e.g., the strength of resting-state networks such as DMN and same-sized random networks?*

Response: We thank the reviewer for this insightful suggestion. To address this, we conducted additional analyses comparing the predictive performance of the full neural signature with models based on simpler feature representations. Specifically, we constructed models using connectivity features within individual canonical networks (e.g., default mode and attention networks). These benchmark models showed variable performance (AUC = 0.62-0.85) in discriminating sleep-deprived from rested states, generally lower than the full model (AUC = 0.91). While some individual networks achieved relatively high performance, none matched the performance of the full model. These results suggest that, although certain networks carry meaningful information about sleep deprivation, the highest predictive accuracy is achieved by integrating distributed connectivity patterns across multiple systems. We have added these analyses to the Supplementary Information and clarified this point in the main manuscript as below.

"We further compared the whole-brain model with models based on connectivity within individual canonical networks. These network-restricted models showed variable performance (AUC = 0.62–0.85, Supplementary Fig. S3) that was generally lower than the whole-brain model (AUC = 0.91), suggesting that integrating distributed connectivity provides improved predictive performance."

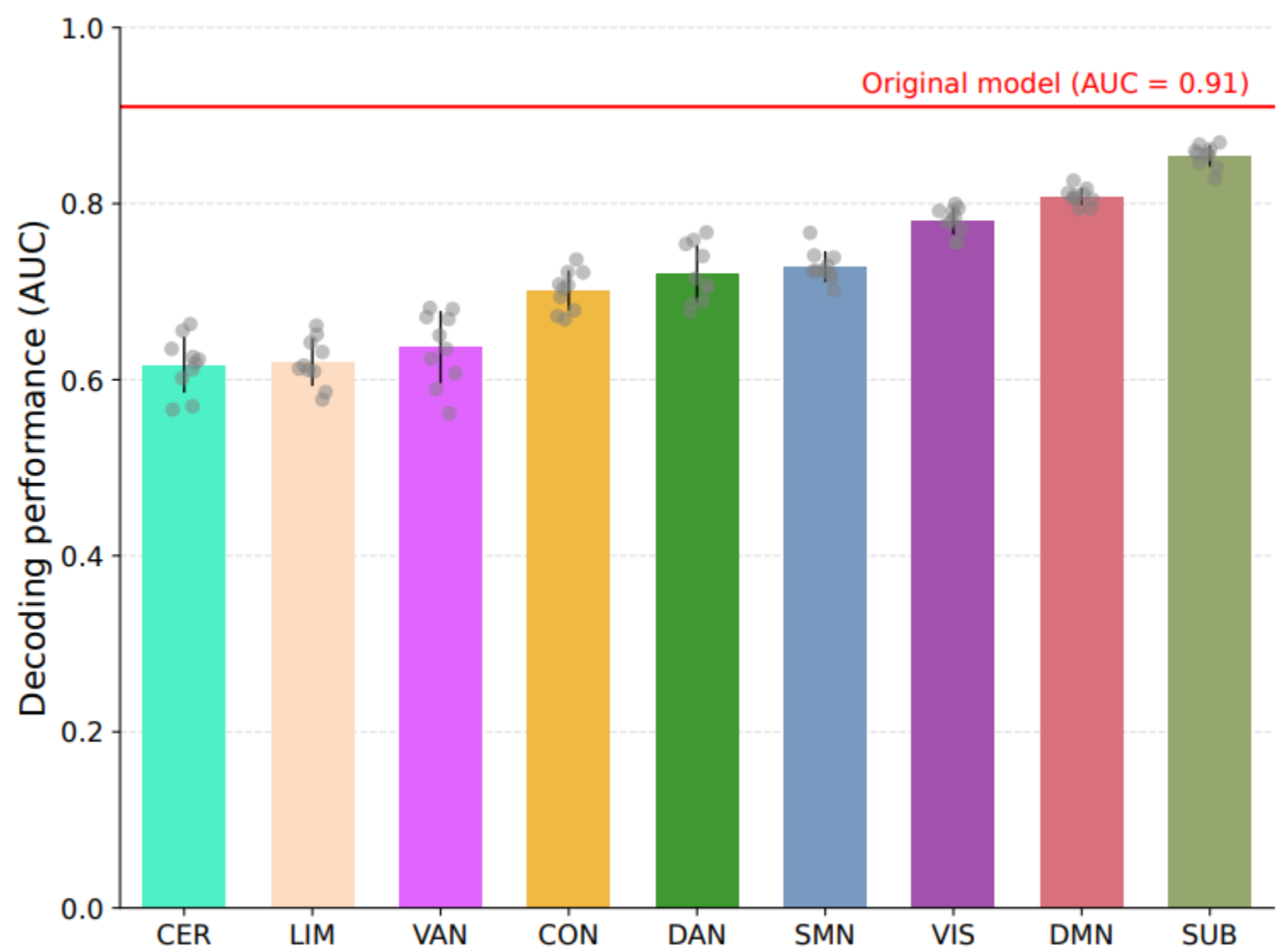

Supplementary Fig. 2. Classification analysis based on functional connectivity within individual canonical networks. To evaluate whether the predictive performance of the neural signature depends on distributed connectivity patterns, we conducted additional analyses comparing the whole-brain model with models based on reduced feature sets. Specifically, for each canonical network, we constructed classifiers using only within-network functional connections. While some individual networks achieved relatively high classification performance, none matched the performance of the whole-brain model.

Comment: *I am interested in hearing more about what specific construct the authors think their model captures. Is it sleep deprivation per se or could it be something related to lack of sleep, such as low mood, low arousal or alertness, stress, frustration, effortful attempts to stay still and awake in the scanner, etc.? It could increase the theoretical impact of the results to test this by, for example, relating sleep model predictions to other measures and relating other neural signatures (e.g., of negative mood, arousal [https://journals.plos.org/ploscompbiol/article/authors?id=10.1371/journal.pcbi.1012994], or alertness [https://elifesciences.org/articles/62376]) to sleep.*

Response: We thank the reviewer for raising this important conceptual question. We agree that sleep deprivation is associated with multiple interrelated processes including but not limited to

reduced alertness, altered arousal, and mood fluctuations. Thus, it is critical to clarify what construct the neural signature reflects.

Our interpretation is that the neural signature identified here is best understood as a state marker of acute sleep loss, reflecting a composite of sleep-related processes rather than a single underlying construct. This interpretation is supported by the within-subject sleep manipulation results (datasets 2–3), where the decoder consistently distinguishes the same individuals in sleep-deprived versus rested states, indicating sensitivity to state changes induced by sleep loss.

To further probe the nature of this state, we conducted an exploratory analysis using an independent dataset with continuous ratings of emotional arousal and valence (Chang et al., 2021, Ke et al. 2025; n = 35). We found that PoSD values were higher during high-arousal compared to low-arousal segments, whereas no clear differences were observed between high- and low-valence segments. Although these results should be interpreted cautiously given the modest sample size, they suggest that the neural signature may be sensitive to arousal-related fluctuations but is not driven by affective valence.

Together with the UK Biobank analyses showing specificity to sleep duration rather than other behavioral measures, these findings indicate that the neural signature is not reducible to general affective or behavioral states (e.g., mood), but instead reflects a sleep-loss–related neural state with some sensitivity to arousal. We have added the following content to the manuscript to clarify this interpretation.

"The neural signature identified here is best interpreted as a state marker of acute sleep loss, reflecting a composite of sleep-related processes rather than a single psychological construct. Sleep deprivation is known to affect multiple domains, including arousal, alertness, and affect, which are inherently interrelated. In an exploratory analysis using an independent movie-watching dataset[73,74], we observed that the expression of the signature was higher during high-arousal compared to low-arousal periods, but did not differ across affective valence, suggesting sensitivity to arousal-related fluctuations rather than emotional valence. Together with the UK Biobank analyses showing specificity to sleep duration rather than other behavioral measures, these findings indicate that the signature is not reducible to general affective states, but instead reflects a sleep-loss–related neural state with some sensitivity to arousal. Future work integrating direct behavioral and physiological measures will be important for further disentangling these components."

Reference:

73. Chang, L. J. *et al.* Endogenous variation in ventromedial prefrontal cortex state dynamics during naturalistic viewing reflects affective experience. *Sci. Adv.* **7**, eabf7129 (2021).

74. Ke, J., Song, H., Bai, Z., Rosenberg, M. D. & Leong, Y. C. Dynamic brain connectivity predicts emotional arousal during naturalistic movie-watching. *PLOS Comput. Biol.* **21**, e1012994 (2025).

In addition, the following content was added to the Supplementary Information:

"To provide initial insight into the psychological processes reflected by the neural signature, we conducted an exploratory analysis using the Friday Night Lights (FNL) dataset (Chang et al., 2021; *n* = 35). In this dataset, participants underwent fMRI while watching a movie, and continuous arousal and valence ratings were obtained from a separate group of participants (Ke et al., 2025).

For each participant, the continuous arousal and valence time series were standardized and then averaged across participants to generate group-level arousal and valence ratings. For each dimension separately, time points were divided into high and low segments using a median split, with time points above the median assigned to the high condition and those below the median assigned to the low condition. Whole-brain functional connectivity matrices were then estimated separately for the high and low segments for each participant. The trained sleep-deprivation decoder derived from Dataset 1 was then applied to each connectivity matrix to obtain probability of sleep deprivation (PoSD) scores for the high- and low-arousal segments and for the high- and low-valence segments.

To test whether PoSD differed between conditions, we fitted linear mixed-effects models with condition (high vs. low arousal, or high vs. low valence) and framewise displacement (FD) as fixed effects and participant as a random intercept. After controlling for FD, PoSD scores were significantly higher during high-arousal than low-arousal segments, suggesting that the neural signature was more strongly expressed during moments of elevated arousal ($b = 0.074$, $z = 3.244$, $p = 0.001$, 95%CI = [0.029, 0.119]). By contrast, no significant difference in PoSD was observed between high- and low-valence segments ($b = -0.016$, $z = -1.079$, $p = 0.281$, 95%CI = [-0.044, 0.013])."

(4) Neural patterns under other sleep manipulations

Comment: *I did not fully follow the significance of the analyses in this section. What is the motivation for testing effects on features with positive vs. negative models weights separately?*

Response: The motivation for separating positive and negative predictive weights was to distinguish between connectivity patterns that contribute to classifying the brain as "sleep deprived" (positive weights) versus those that contribute to classifying the brain as "well rested" (negative weights). This separation allows us to test whether these two sets of features show differential sensitivity to sleep manipulations. For example, we observed that positive weights were relatively stable after recovery sleep, whereas negative weights showed greater restoration, suggesting asymmetry in how different network features respond to recovery. We have now clarified this rationale in the Results section.

"We separated positive versus negative predictive weights to distinguish connections that increased the likelihood of classifying an individual as sleep deprived versus those that increased the likelihood of classifying an individual as well rested. This allowed us to test whether these distinct sets of features were differentially modulated by recovery sleep or partial sleep restriction."

Methods.

Comment: *In the discovery dataset, data from different tasks (not rest) were concatenated to create a single functional connectivity matrix per person. Did task performance and/or the relative ratio of different tasks differ between groups? Are predictions robust to these potential confounds? If task performance did differ between sleep conditions and groups, this makes a specificity test showing that the sleep models do not predict cognitive performance better than they predict sleep even more important.*

Response: We thank the reviewer for raising this important point. We agree that differences in task performance or task composition could potentially influence functional connectivity estimates in the discovery dataset. In the discovery dataset, all participants completed the same task structure with identical task composition across conditions, ensuring that the relative proportion of task types was matched between groups. In addition, part of the paradigm (fear conditioning and extinction) involved passive stimulus exposure, which minimizes performance-related

variability. Together, these features reduce the likelihood that group differences in connectivity are driven by differences in task composition or engagement.

Importantly, the trained decoder generalized robustly to multiple independent validation datasets (datasets 2–4), many of which were acquired during resting-state fMRI. In these datasets, the decoder successfully distinguished sleep-deprived from rested states within individuals. This cross-context generalization suggests that the neural signature is not dependent on specific task structure or performance differences, but instead reflects a sleep-loss–related neural state that is expressed across different cognitive contexts. We have clarified this point in the revised manuscript.

"All participants completed identical task structures with matched timing and composition across conditions, minimizing potential confounds from task differences. In addition, part of the paradigm involved passive stimulus exposure, reducing performance-related variability."

Dear Editor and Reviewers,

We herein submit our revised manuscript “A neural signature of sleep deprivation in the human brain” to be considered for publication in Nature Communications. We are grateful to the reviewers and editor for your careful read of the paper and your insightful and constructive comments. We have revised the manuscript in response to all of the comments. Below, we provide a point-by-point response to each comment and indicate the corresponding changes made in the manuscript (marked in red).

Reviewer #1 (Remarks to the Author):

Comment: Thank you for the reply. Using a unified color scheme to preserve visual clarity is understandable. However, given a trend of significant group differences observed in dataset 3 ($p=0.09$), I recommend reporting separate descriptive values for PoSD changes in patients and controls in addition to t values.

Response: We thank the reviewer for this helpful suggestion. We have now added separate descriptive values for PoSD changes in patients and controls in datasets 2 and 3, in addition to the between-group t tests.

"We found no significant group differences in dataset 2 ($t(66) = -1.38$, $p = 0.172$; mean±s.d.: controls, 0.42 ± 0.28; patients, 0.53 ± 0.32) and dataset 3 ($t(52) = 1.69$, $p = 0.098$; controls, 0.45 ± 0.16; patients, 0.30 ± 0.27)"

Reviewer #2 (Remarks to the Author):

Comment: I appreciate the authors' clarifications, however Table S1 (which should be included in the main text because it conveys critical information to understand the study) clearly shows that the majority of validation datasets used resting state fMRI (all but one) and/or within-subject design. In these conditions, how can the authors justify the use of a task-based fMRI between-subject dataset as their training model ? This should be explicitly discussed. Why not using a resting-state dataset for training as this may be more generalizable?

Response: We thank the reviewer for this important comment. We agree that Table S1 contains critical information for interpreting the study design and have moved it to the main manuscript.

We have also added an explicit discussion justifying the use of dataset 1 for model training. Prior work has shown that large-scale functional connectivity patterns are highly similar across task and resting states, and dataset 1 used random assignment of healthy participants to sleep conditions. Importantly, the resulting decoder generalized successfully to multiple independent resting-state datasets with within-subject sleep manipulation designs, suggesting that the identified pattern is not specific to the training paradigm. Because dataset 1 did not include resting-state fMRI, the available resting-state datasets were reserved for independent validation. The following paragraph was added to the revised manuscript.

"The neural signature was developed using task-based fMRI data from a randomized between-subject discovery dataset and subsequently generalized to independent resting-state datasets with within-subject sleep manipulation designs. Functional connectivity estimated during task performance captures coordinated interregional fluctuations and has been shown to preserve large-scale network organization observed during resting-state[75–78]. Moreover, random assignment of healthy participants to sleep conditions in the discovery dataset reduced confounding by pre-existing individual differences. Although dataset 1 did not include resting-state fMRI, the successful generalization of the task-based decoder to independent resting-state datasets suggests that the identified pattern is not specific to the training task or study design, but instead reflects sleep-loss-related connectivity changes that generalize across contexts."

References:

75. Cho, J. W., Korchmaros, A., Vogelstein, J. T., Milham, M. P. & Xu, T. Impact of concatenating fMRI data on reliability for functional connectomics. *NeuroImage* **226**, 117549 (2021).

76. Gordon, E. M. *et al.* Precision functional mapping of individual human brains. *Neuron* **95**, 791–807 (2017).

77. Cole, M. W., Bassett, D. S., Power, J. D., Braver, T. S. & Petersen, S. E. Intrinsic and Task-Evoked Network Architectures of the Human Brain. *Neuron* **83**, 238–251 (2014).

78. Gratton, C. *et al.* Functional Brain Networks Are Dominated by Stable Group and Individual Factors, Not Cognitive or Daily Variation. *Neuron* **98**, 439-452.e5 (2018).

Reviewer #3 (Remarks to the Author):

Comment: The revision thoroughly and thoughtfully addresses my comments. As one final suggestion, the authors may want to consider moving the arousal findings to the results. They are informative about what process(es) model predictions may reflect and it seems out of place to introduce new results in the discussion. - Monica Rosenberg

Response: We thank the reviewer for the positive feedback and helpful suggestion. Following the reviewer's recommendation, we have moved the arousal/valence analyses to the Results section and revised the Discussion accordingly. The following sentences were added to the Results section.

"To further explore the processes reflected by the neural signature, we conducted an exploratory analysis in an independent movie-watching fMRI dataset with continuous ratings of emotional arousal and valence[48,49]. PoSD scores were higher during high-arousal than low-arousal periods, whereas no significant difference was observed between high- and low-valence periods (**Supplementary Results**). These findings suggest that the neural signature may be more sensitive to arousal-related processes than to affective valence."

References:

48. Chang, L. J. *et al.* Endogenous variation in ventromedial prefrontal cortex state dynamics during naturalistic viewing reflects affective experience. *Sci. Adv.* **7**, eabf7129 (2021).

49. Ke, J., Song, H., Bai, Z., Rosenberg, M. D. & Leong, Y. C. Dynamic brain connectivity predicts emotional arousal during naturalistic movie-watching. *PLOS Comput. Biol.* **21**, e1012994 (2025).
